# Dynamics of early gut microbiota maturation in extremely preterm infants and neurodevelopment at 2 years of age in a probiotic intervention trial

**DOI:** 10.1016/j.isci.2026.115802

**Published:** 2026-04-20

**Authors:** Thomas Abrahamsson, Erik Wejryd, Meritxell Pujolassos, M. Luz Calle, Eva Sverremark-Ekström, Maria C. Jenmalm, Magalí Martí

**Affiliations:** 1 Department of Biomedical and Clinical Sciences, Linköping University, Linköping, Sweden; 2 Crown Princess Victoria Children’s Hospital, University Hospital, Linköping, Sweden; 3 Clinical Department of Pediatrics, Vrinnevi Hospital, Norrköping, Sweden; 4 Department of Bioscience, University of Vic – Central University of Catalonia, Vic, Spain; 5 Institut de Recerca i Innovació en Ciències de la Vida i de la Salut a la Catalunya Central (IRIS-CC), Vic, Spain; 6 Department of Molecular Biosciences, The Wenner-Gren Institute, Stockholm University, Stockholm, Sweden

**Keywords:** Health sciences, Medicine, Microbiology, Neuroscience

## Abstract

Preterm birth is associated with a high risk of long-term neurological deficits. Although research underscores the role of the gut microbiome in the gut-brain axis, the mechanisms of neurodevelopmental impairment remain elusive. In this prospective observational study (PROPEL), we evaluated whether early gut microbiota development and *Limosilactobacillus reuteri* supplementation to extremely preterm infants with extremely low birth weight (EPT-ELBW) are associated with neurodevelopment at 2 years. Gut microbiota was characterized by 16S sequencing, and neurodevelopment was assessed by Bayley-III score. Microbiota composition constellations and lower microbial diversity, but not single bacteria, are associated with impaired neurodevelopment. Microbial maturation over the first month was discriminative for motor development, with higher abundance of *E*. *coli* and *Enterococcus* relative to *Cutibacterium* associated with impairment. *L*. *reuteri* supplementation did not seem to affect neurodevelopment via the gut microbiome. In conclusion, dynamics of gut microbiota maturation during early life may impact neurodevelopment in EPT-ELBW infants.

## Highlights

• Early gut microbiota maturation is linked to neurodevelopment in EPT-ELBW infants
• Higher gut microbial diversity is related with better Bayley-III outcomes at 2 years
• Microbiota constellations, not single taxa, associate with impaired neurodevelopment
• *L*. *reuteri* supplementation may not influence neurodevelopment via the gut microbiome

## Graphical abstract

**Figure:**
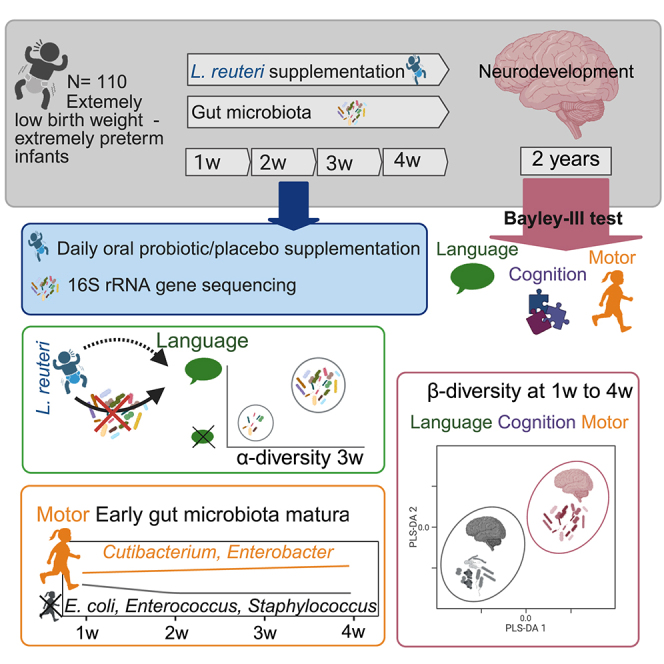

## Introduction

Preterm birth is a leading cause of infant mortality and often results in morbidity and disability among survivors.^1^ While mortality rates have decreased during the last decade,^2^ the survivors often develop long-term neurological deficits with cognitive and behavioral problems.^3^^,^^4^^,^^5^ The severity of the impairment is inversely associated with gestation age, where extremely preterm (EPT; born before 28 completed gestational weeks [gws]) infants are at higher risk of developing neurodevelopment disabilities.^6^^,^^7^ The long-term neurological disabilities in adolescence and adulthood are mainly cognitive impairment, learning and language disorders, attention deficit hyperactivity disorder, intellectual disability, and autism.^4^^,^^6^^,^^8^

Neurodevelopmental impairment after EPT birth is common.^5^ The French Epipage-2 trial found that 50% of infants born in gw 24–26 exhibited a moderate or severe neurodevelopmental impairment (<2 standard deviation [SD] below reference). The communication domain was most severely affected.^9^ In British 3-year-old children born in gw 22–26, a moderate or severe impairment was found among 25% of the survivors.^10^ Swedish infants born before gw 27 assessed at 2.5 years corrected age had moderate to severe impairment in 11% (cognition) and 16% (language) of the cases.^7^ A Japanese study of infants born 2003–2014 showed that the level of neurodevelopmental impairment among EPT infants was not decreasing over time.^11^

The mechanisms leading to neurodevelopment impairment remain unclear, although during the last decade, it has become apparent that the gut microbiome is a key player regulating the gut-brain axis function.^12^^,^^13^^,^^14^ During the postnatal period, early stages of neurodevelopment occur in parallel with the gut microbiota development,^15^^,^^16^^,^^17^ and increasing evidence indicates that the early life microbiota contributes to the bidirectional gut-brain axis communication and consequent brain development and maturation regulation.^15^^,^^18^^,^^19^^,^^20^ It is during these critical early stages of development, when the gut microbiota composition as well as its maturation in preterm infants differs from the term infants.^15^^,^^21^^,^^22^^,^^23^^,^^24^^,^^25^ Nonetheless, EPT infants remain poorly investigated, although a recent study showed that an aberrant development of the gut-microbiota-immune-brain axis during the first months of life, may be associated with neurophysiological development resulting in brain injury during the neonatal period.^26^

In contrast to EPT, more studies have assessed the effects of probiotic supplementation, mainly *Bifidobacterium* sp, *Lactobacillus* sp, and/or *Limosilactobacillus* (*L*.) *sp* to less immature preterm infants in relation to neurodevelopment and, generally, no effects of probiotic supplementation in relation to neurodevelopment have been observed.^27^^,^^28^ However, Romeo et al.^29^ showed that preterm infants supplemented with *L*. *reuteri* or *Lacticaseibacillus rhamnosus* had lower incidence of abnormal neurological outcomes at 1 year of age, and Jacobs et al.^30^ found preterm infants supplemented with *B*. *infantis*, *B*. *lactis*, and *Streptococcus thermophilus* to have lower incidence of sensorial hearing loss at 2 years corrected age.

Altogether, the results of previous studies suggest that the infant demographics (gestational age at birth, birth weight, study site), the age when gut microbiota was analyzed, time/duration/strain(s) of probiotic supplementation, as well as infant age for neurodevelopment assessment impact the results.^15^^,^^21^^,^^22^^,^^23^^,^^24^^,^^25^^,^^31^^,^^32^^,^^33^^,^^34^ Therefore, it is important to have a good coverage of all different infant cohorts, and thus, more studies including EPT infants are needed. In a previous clinical trial (PROPEL), we have shown that daily supplementation during the neonatal period with *L*. *reuteri* DSM 17938 to EPT infants with extremely low birth (EPT-ELBW), modulates their gut microbiota,^31^ and is associated with improved head growth during the first month of life,^35^ as well as better language development at 2 years corrected age.^36^ Therefore, in this study we sought to evaluate (a) whether modulation of the gut microbiota by *L*. *reuteri* supplementation and (b) microbiota development during the first month of life, are associated with better neurodevelopment at 24 months corrected age, including language development, cognition development, motor-skills development, and overall neurodevelopment impairment (NDI).

## Results

### EPT-ELBW infant cohort

From the 134 infants originally enrolled in the PROPEL-trial, a 2-year follow-up was possible on 110 infants, from which, we had 16S amplicon data from 105 infants (Table 1). The number of infants varied across weeks because fecal samples were unavailable at some time points and follow-up assessments were occasionally incomplete. Among the background characteristics of the infants (Tables 2 and S1), several clinical variables significantly differed between the normal and impairment group, including: gestational age, birth weight, birth length, birth head circumference, bronchopulmonary dysplasia, Apgar at 10 min, as well as inclusion site. Consistently, the infants in the impairment group had the more adverse outcome of the above-mentioned clinical characteristics. Infant sex did not differ between the normal and impairment group. Among the family background variables, multilingualism was associated with higher odds of language impairment, with odds ratios of 3.36 (95% CI, 1.16–9.67) at week 2, 5.25 (95% CI, 1.6–16.9) at week 3, and 6.7 (95% CI, 1.9–24.6) at week 4. The substantial amount of missing data for parental postgraduate education and mother tongue, limits reliable interpretation. Among all the above-mentioned variables (Table S1), only gestational age, inclusion site and multilingualism were considered as confounding factors. Inclusion site at 1 w was significantly associated with alpha-diversity, beta-diversity, and taxa abundance for language, motor, and NDI outcomes (Table S2). At 2 w was significantly associated with alpha-diversity for NDI, and at 3 w and 4 w to taxa abundance for NDI. Gestational age, categorized as above or below the mean, was significantly associated at 1 w with beta-diversity for motor and NDI, and at week 2 and week 4 to taxa abundance for NDI. No associations between multilingualism and microbiome were found.

**Table 1:** Number of infants at each group at 1 week (w), 2 w, 3 w, and 4 w of life

		1 w	2 w	3 w	4 w	Longitudinal
Language	Normal	36	38	35	34	35
	Impairment	31	29	31	27	30
Cognition	Normal	49	48	46	46	46
	Impairment	22	22	23	19	22
Motor	Normal	49	49	46	45	47
	Impairment	13	14	15	13	13
NDI	Normal	48	50	43	44	47
	Impairment	42	42	45	41	41

NDI: neurodevelopment impairment. Longitudinal: number of infants with at least three observations across the four weeks.

**Table 2:** Background and clinical characteristics of the extremely preterm—extremely low birth weight infants from which stool samples collected at week one of life were compared to language outcome at two years corrected age

Variables	Week 1		
	Normal (*n* = 36)	Impairment (*n* = 31)	*p* value^a^
Gestational age, weeks, mean (SD)	25.7 (1.3)	25.2 (1.3)	0.08
Birth weight, g, mean (SD)	790 (123)	682 (123)	**0.001**
Birth weight *Z* score, mean (SD)	−0.8 (1.3)	−1.3 (1.3)	0.10
Birth length, cm, mean (SD)	33.5 (2.2)	31.8 (2.4)	**0.01**
Birth length, *Z* score, mean (SD)	−1,0 (1.7)	−1.7 (1.7)	0.10
Birth head circumference, cm, mean (SD)	23.4 (1.4)	22.5 (1.5)	**<0.001**
Birth head circumference *Z* score, mean (SD)	−0.7 (0.7)	−0.8 (0.7)	0.40
Apgar at 10 min, mean (SD)	8.3 (2)	7.5 (2)	0.07
SGA, n (%)	28 (78)	23 (74)	0.78
Female sex, n (%)	17 (47)	17 (55)	0.53
Infants from multiple pregnancy, n (%)	14 (39)	8 (26)	0.26
Prenatal steroids administered, n (%)	35 (97)	31 (100)	1.00
Chorioamnionitis, n (%)	9 (25)	6 (19)	0.58
Cesarean section, n (%)	25 (69)	19 (61)	0.48
Inclusion site			
Linkoping, n (%)	10 (28)	18 (58)	**0.02**
Stockholm, n (%)	26 (72)	13 (42)	

**Neonatal hospital care**			

Bronchopulmonary dysplasia, n (%)	19 (53)	24 (77)	**0.04**
Sepsis, n (%)	7 (19)	10 (32)	0.23
Retinopathy of prematurity, grade 3–5, n (%)	4 (11)	9 (29)	0.06
Intraventricular hemorrhage, grade 3–4, n (%)	3 (8)	3 (10)	1.00
Necrotizing enterocolitis, grade 2–3, n (%)	1 (3)	2 (6)	0.44
Days on antibiotics week before sampling, median (IQR)	7 (0)	7(0)	0.51

**Family background**			

Smoking in family at inclusion, n (%)	0 (0)	5 (16)	**0.02**
Parents with postgraduate education			
At least one, n (%)	17 (47)	10 (32)	0.28
None, n (%)	7 (19)	11 (35)	
No data, n (%)	12 (33)	10 (32)	
Mother tongue			
Swedish or Scandinavian, n (%)	21 (58)	15 (48)	0.461
Other, n (%)	5 (14)	8 (26)	
No data, n (%)	10 (28)	8 (26)	
Multiple languages spoken at home			
No n (%)	27 (75)	18 (58)	0.072
Yes, n (%)	7 (19)	13 (42)	
No data, n (%)	2 (6)	0	

Significant p values (<0.05) are marked in bold.

a *t* test for independent samples for continuous data. χ^2^ test for categorical data (Fisher’s exact test when appropriate).

### *L*. *reuteri* supplementation and neurodevelopment

For this reduced nested cohort, with matched data available for the microbiota composition and Bayley-III test, we found no associations between *L*. *reuteri* supplementation and neurodevelopment, the latter being assessed as normal vs. impaired (Table S3). Consequently, we did not observe any effect of the microbiota in mediating the association between *L*. *reuteri* DSM 17938 supplementation and neurodevelopment outcomes when categorized as normal and impairment groups (Table S4). Nonetheless, *Lactobacillus* was consistently significantly more abundant in the *L*. *reuteri* group, while *Finegoldia* was marginally more abundant in the placebo group at week 4 for the motor outcome. *L*. *reuteri* supplementation was significantly associated with higher alpha-diversity at week 1 and week 2 (Table S3), and for the placebo group, higher alpha-diversity at week 3 was associated to normal NDI outcome at 2 years of age (Table S5). However, alpha-diversity showed no mediating effect on the neurodevelopment outcomes (Table S6). Given the lack of significant associations between the *L*. *reuteri*/placebo groups and language development score, cognition development score, motor development score as well as overall neurodevelopment, we proceeded to the investigation of potential associations between the gut microbiota and neurodevelopment outcomes without taking into consideration whether the infants received *L*. *reuteri* or placebo supplementation.

### Microbial taxa in relation to neurodevelopment outcomes

**Figure 1:**
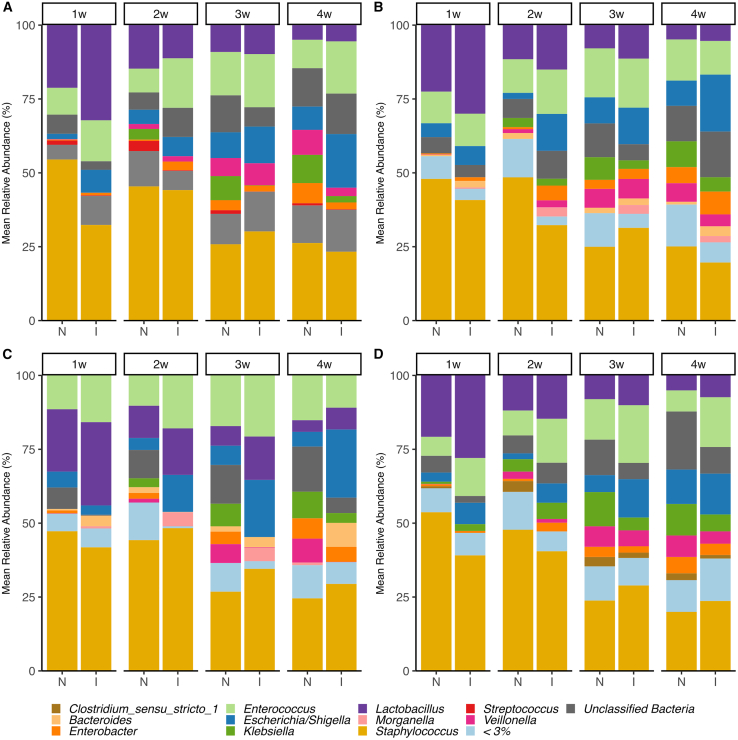
Taxonomic composition, at genus level, of the gut microbiota in ELBW-EPT infants with normal (N) or impaired (I) neurodevelopment at 2 years of age (A) Language development, (B) cognitions development, (C) motor development, and (D) overall neurodevelopment. less than 3% includes all genera with a relative abundance of less than 3% across all samples within each dataset. See Table 1 for the number of samples in each group.

The gut microbiota of these EPT-ELBW infants during the first month of life was dominated by a few genera, including *Staphylococcus*, *Enterococcus*, *Escherichia (E*.*) coli*, *Klebsiella*, and *Lactobacillus* (Figure 1). These dominant genera were overall, equally represented in both normal and impairment groups, although minor descriptive differences were observed at specific timepoints. For example, at 1 w the normal language development group had slightly higher relative abundance of *Staphylococcus* and less of *Lactobacillus*; at 4 w, subtle shifts occurred, with the impairment group showing higher relative abundance of *E*. *coli*, while the normal groups exhibited increased representation of *Klebsiella* and *Veillonella*. However, no significant differential abundant bacteria (at genus level and prevalence threshold of ≥10%) were identified between the normal and impaired groups for each timepoint, as assessed with ANCOM-BC, adjusting for the above-mentioned confounding factors, with one exception, *Klebsiella* had significantly lower abundance in the language impairment group at 3 w compared to the normal group (log fold change = −2.24, q-value = 0.03). Moreover, in some cases for cognition and motor development certain genera were exclusively found in the normal group (Table 3). We further explored whether there was a difference in Bayley-III scores associated to colonization of these bacteria only present in the normal development group, and we found that infants colonized with *Finegoldia* at 3 w had higher Bayley-III score for cognition (*p* value = 0.02, q value = 0.14).

**Table 3:** Prevalence and relative abundance of genera only present at the normal group in respect to the impaired group

Outcome	Timepoint	Genera	Prevalence (N)	Prevalence (%)	Relative abundance (%)	Mann-Whitney U Test (*p*-value)
Cognition	3 w	*Finegoldia*	7	15	0.8	0.02 (q value: 0.14)
	4 w	*Kluyvera*	8	17	1.9	0.36
Motor	1 w	*Enterobacter*	9	18	2.6	0.43
		*Cutibacterium*	9	18	0.1	0.28
	2 w	*Acinetobacter*	7	14	2.1	0.39
		*Finegoldia*	7	14	0.4	0.48
	3 w	*Finegoldia*	7	15	0.8	0.34

Prevalence (N): N genera within the normal group.

Prevalence (%): prevalence in % within the normal group.

Relative abundance: for the normal group without previously filtering to 10%.

Mann-Whitney U test: Bayley-III scores in infants with and without colonization of specific genera. The q value was obtained after Benjamini & Hochberg correction.

### Higher microbial richness at 3 weeks of life associated with language development

**Figure 2:**
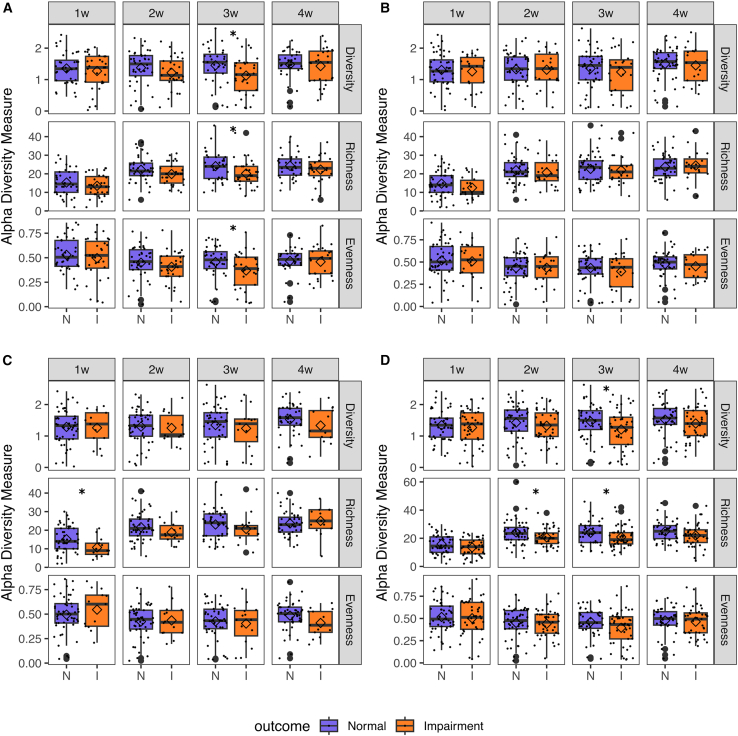
Gut microbiota alpha-diversity of ELBW-EPT infants with normal (N) or impaired (I) neurodevelopment at 2 years of age (A) Language development, (B) cognitions development, (C) motor development, and (D) overall neurodevelopment. Data are represented as boxplots (median with 25% and 75% percentiles and 1.5× the interquartile range; diamond shape depicts the mean) showing the a-diversity (Shannon index), richness (observed ASVs), and evenness (Pielou’s evenness index) from 1 week to 4 weeks of life. ∗*p* < 0.05 by Student’s *t* test. See Table 1 for the number of samples in each group. See also Figure S1.

We also assessed whether the neurodevelopment was associated with differences in the community structure (alpha diversity). Overall, there was a tendency toward lower alpha-diversity during the first month of life in the impairment groups (Figure 2). For language development, a decrease in richness (b = −0.08), diversity (b = −0.89), and evenness (b = −2.78) at 3 w of age, was significantly associated with higher probability of developing language impairment at 2 years of age (Figure S1). A decrease in diversity (b = −0.74) at 3 w was also significantly associated with increased probability of developing overall NDI (Figure S1).

### Distinct microbial community composition between normal and impaired outcomes

**Figure 3:**
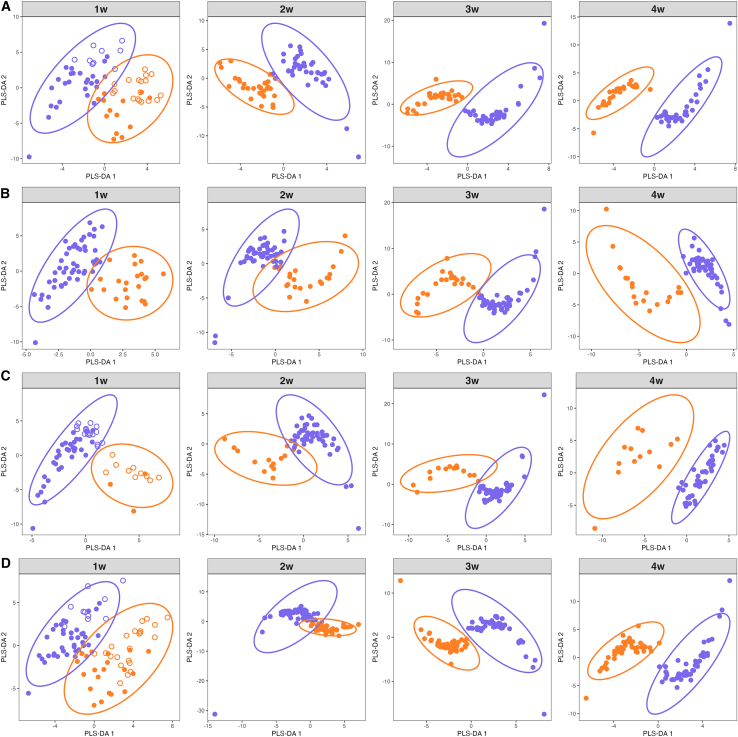
Partial least-square-discriminant analysis (PLS-DA) at ASV-level of sample distribution (A) Language development, (B) cognitions development, (C) motor development, and (D) overall neurodevelopment. Orange circles denote impairment outcome group and purple circles denote normal outcome group. At 1 week (w), the open and filled circles denote inclusion site. See Table 1 for the number of samples in each group.

Differences in beta-diversity, at ASV level, between the normal and impaired groups at each timepoint were assessed with NMDS and ANOSIM, revealing no significant difference in the microbial community composition between the groups (Figure S2). A supervised PLS-DA was performed to model the community at ASV level, which revealed distinct microbial community compositions among the normal and impaired groups for each timepoint (Figure 3). Because the separations were mainly driven by component 1, we selected the main ASVs contributing to component 1 based on their loading weight, in order to characterize the ASVs associated to each group (Table S7). The most important ASVs contributing to both groups were assigned to *Staphylococcus* sp., *Lactobacillus* sp., and other taxa among the dominant ones. Nonetheless, although a sparse PLS-DA was further applied to identify a minimal discriminate signature, no well-performing models were obtained. A PLS-DA at genus-level did not reveal any clear separation between the two groups for any outcome, thus confirming the lack of identification of key genera discriminative of normal or impaired language, cognition, motor, and overall, NDI at a given timepoint (data not shown). These results suggest it may be several bacteria in conjunction that are important for the neurodevelopment instead of individual taxa.

Table S7. Loading weights of the ASVs on PLS-DA component 1, related to Figure 3

### Microbiota maturation associated with neurodevelopment

**Figure 4:**
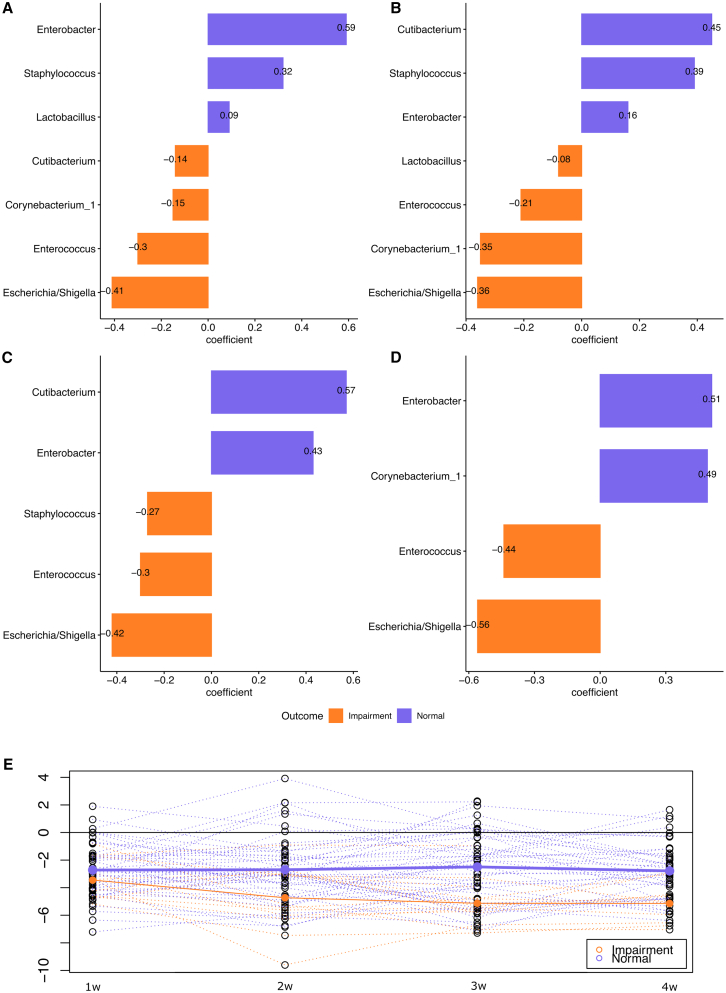
Microbial signature that discriminates between the normal and impaired groups (A) Language development, (B) cognitions development, (C) motor development, and (D) overall neurodevelopment. (E) Microbial signature trajectories depicting the mean relative abundance of the microbial signature discriminative for normal and impairment group for motor development from 1 week (1 w) of life to 4 weeks (4 w) of life. See Table 1, for the number of samples in each group. The microbial signatures were identified using the function coda_glmnet_longitudinal().

The development of the gut microbiota over the first month of life was analyzed using the *coda4microbiome* to model how the development dynamics of the microbiota differed between the two groups, and to identify the group of taxa that best discriminates between the two groups. For each development outcome, we identified a microbial signature whose trajectories over the first month life differed between the normal and impaired group (Figure 4). A microbial signature consists of the relative abundances of two groups of taxa, one associated to normal development (GroupN) and the other to impaired development (GroupI), and the trajectories of the microbial signature over time depict the abundance of a group relative to the abundance of the other group. Based on the accuracy measures, the model for motor development was the best one (mean cross-validation [cv] AUC = 0.75) in contrast to the cognition development model which had a mean cv-AUC of only 0.49 (Table S8).

The microbial signature for motor development consisted of *Cutibacterium* and *Enterobacter* (GroupN), and *Escherichia/Shigella*, *Enterococcus*, and *Staphylococcus* (GroupI; Figure 4C). Infants with impaired motor development had higher mean relative abundance of *Escherichia/Shigella*, *Enterococcus*, and *Staphylococcus* in relation to the relative abundance of the taxa in GroupN. In contrast, infants with normal motor development had higher mean relative abundance of *Cutibacterium* and *Enterobacter*. The differences in relative abundance between the taxa in each group (GroupN and GroupI) increased at week 2 due to an increase in relative abundance of the taxa in GroupI with respect to GroupN, which was stable over the month (Figure 4E).

The microbial signature for language development was composed of *Enterobacter*, *Staphylococcus*, and *Lactobacillus* (GroupN), although *Lactobacillus* contributed very little to the signature, and *Escherichia/Shigella*, *Enterococcus*, *Corynebacterium*, and *Cutibacterium* (GroupI; Figure 4A), with *Escherichia/Shigella* and *Enterococcus* being the main contributors for GroupI. The differences in relative abundance between the taxa in each group (GroupN and GroupI) increased at week 3 due to progressive increase in relative abundance of the taxa in GroupI with respect to GroupN, which was stable over the month (Figure S3).

For cognition, the main taxa contributing to the GroupN were *Cutibacterium and Staphylococcus*, and the main taxa contributing to the GroupI were *Escherichia/Shigella* and *Corynebacterium* (Figure 4B). The microbial signature for NDI consisted of *Enterobacter* and *Corynebacterium* (GroupN) vs. *Escherichia/Shigella* and *Enterococcus* (GroupI), (Figure 4D).

## Discussion

Although daily supplementation with *L*. *reuteri DSM 17938* during the neonatal period (until postmenstrual week 36 + 0) was associated with improved language score at 24 months corrected age in this cohort in a previous study,^36^ there were no difference when the infants were classified as having a normal or impaired neurodevelopment, which is in line with previous meta-analyses reporting no overall differences in neurodevelopment following probiotic or prebiotic supplementation.^37^ Consequently, we could not identify a mediation mechanism of the *L*. *reuteri* supplementation via the gut microbiome. Therefore, we hypothesize that *L*. *reuteri* may exert an effect on the central nervous system via a direct effect on the intestinal mucosa and the enteral nervous system,^38^ through for example the production of γ-aminobutyric acid,^39^ regulation of tryptophan/serotonin metabolism^40^ or by promoting biopterin metabolite (BH4),^41^ as reported in animal models.

The fact that we could not identify any specific taxa to be discriminative between EPT-ELBW infants with normal vs. impaired neurodevelopment could be explained by the unique microbiota composition of these infants, which is constrained by the distinct conditions of preterm infants, including, among other, antibiotic treatment, postmenstrual age, delivery mode, delayed enteral feeds, breast milk, and neonatal intensive care unit stay.^15^^,^^22^^,^^42^^,^^43^^,^^44^ Indeed, the taxonomic profile in our dataset is characteristic of the preterm infants during early life, which is typically dominated by *Staphylococcous*, *Entetrococcus*, *E*. *coli*, and *Klebsiella*, all potential pathobionts, and *Lactobacillus*.^22^^,^^24^^,^^25^^,^^26^^,^^31^^,^^42^^,^^45^^,^^46^^,^^47^ Due to the proactive care of EPT-ELBW infants in Sweden, our cohort consisted of more immature infants than the other cohorts assessing the relationship between gut microbiome and neurodevelopment.^47^^,^^48^^,^^49^ The rate of antibiotic treatment was very high during the first 4 weeks of life,^50^ which may explain why our study did not confirm the association between low abundance of *Bifidobacterium* during the neonatal period and neurodevelopmental impairment reported in previous trials,^48^^,^^49^ despite exclusive feeding with breast milk due to 100% coverage of donor milk banks during the neonatal period. Pasteurized donor milk favors an intestinal microbiome more similar to mother’s own milk than formula,^51^ although bacteria of unpasteurized mother’s own milk contribute better to the gut microbiome of preterm infants.^52^ Another explanation for the lack of significant individual taxa in discriminating between both groups, may be that the low gestational age and other exposures such as hypoxia, hypotension, hyperglycemia, and poor nutrition of the EPT infants may be more important for neurodevelopment than an individual bacterium.

Nonetheless, an increase in absolute abundance of *Klebsiella* at 4 weeks of age in EPT infants has been associated with brain damage severity assessed with magnetic resonance imaging during the neonatal period.^26^ Enrichment of *E*. *coli* at 4 weeks of age in EPT infants has been associated with higher ages and stages questionnaire scores at 2 years of age, whereas enrichment in *Staphylococcus* has been associated with poorer neurodevelopment.^46^^,^^47^ Assessing actual clinical outcome later in infancy using the Bayley tool, rather than using radiologic findings or questionnaires, is a strength in this study. A limitation is the early assessment at 2 years of age, although 2 years of age is used in the Swedish follow-up after EPT birth, and widely internationally, as a benchmark for early detection of cerebral palsy, severe cognitive delay, and major sensory impairments.^53^ Executive, attention, and neuropsychiatric difficulties often emerge at school age.^54^ Later follow-up would be more accurate for predicting long-term development and reveal subtle impairments and autism, which may be reduced by childhood probiotic supplementation.^55^ Additionally, the methodology and data analysis *per se* may also be a source of discrepancies in the results. In our study we applied the ANCOM-BC to assess taxa that may be differently abundant between groups,^56^ while the comparative study used a completely different approach.^26^ Moreover, when investigating ecological community studies, it is crucial to differentiate between true biological associations and statistical associations inferred from compositional microbiome data, i.e., compositional microbiome data.^57^ To address this issue, we have applied a compositional data analysis (CoDA) framework, which accounts for these inherent properties of compositional data and focuses on inter-taxa relationships, yielding more robust and biologically meaningful interpretations of the microbial community structure and dynamics.^58^

In adults, gut microbiota alpha-diversity is generally positively associated to health^59^^,^^60^ and lower diversity has been related to psychiatric disorders.^61^ Accordingly, in this study we found that the EPT-ELBW infants with normal language development as well as a normal overall NDI had higher alpha-diversity throughout the first month of life. However, other studies on term infants have reported higher diversity to be related to adverse neurocognitive outcomes. Specifically, higher diversity at 1 year of age has been negatively associated with cognitive performance at 2 years of age^20^ and with behavior problems.^62^ Higher diversity at three days of life has also been linked to lower communication scores at 6 months of age in term-infants born small for gestational age.^19^ Nonetheless, all these studies differ in the timepoints when diversity and neurocognitive outcomes are associated. Differences in sequencing technologies and data analyses may also be a source for discrepancies.^63^ However, it is worth noticing that term-infants have higher diversity than preterm infants,^24^^,^^25^^,^^47^ and thus, we hypothesize that the higher diversity in our cohort may resemble the lower diversity in term infants. This would indicate that there may be certain gut microbiota constellations beneficial for the neurodevelopment in infants, which in turn may be specific to infant cohort. Indeed, the distinct gut microbiota composition observed between the normal and impairment groups supports this hypothesis.

The importance of the composition of the gut microbiota community was further sustained by the longitudinal analysis which revealed, for each development outcome, a microbial signature whose trajectories differed over the first month of life. Longitudinal microbiome studies strengthen causal inference by capturing the dynamics of microbial communities, including maturation, stability, responses to antibiotic exposure, and changes preceding or following disease onset, while accounting for both inter- and intra-individual variation.^64^^,^^65^^,^^66^ The development of the gut microbiota composition and function from early-life has been shown to be dynamic and may stabilize between 3 to 5 years of age,^42^^,^^67^^,^^68^^,^^69^ with microbiome composition trajectories during the first month potentially associated with neurodevelopmental outcomes in early childhood.^37^ For term infants, the development phases start with and immature *E*. *coli-*dominated community to mature toward a *Bacteroides*-dominated community.^68^ For preterm infants, four distinct phases of microbiota development have been described from birth up to 60 days postnatal age, being characterized by a transition from *Staphylococcus*-dominated community, to *Enterococcus-*dominated, to then *Enterobacter-*dominated, and ultimately *Bifidobacterium-*dominated community.^22^ Our study time span falls in the first phase (birth to 35 weeks postmenstrual age) and accordingly, the gut microbiota during the four first weeks of life was a *Staphylococcus*-dominated community. *Staphylococcus* contributed to the impairment signature for the motor development (the model with sufficient discriminative power), while it was associated with the normal development signatures in the other models. Notably, a recent study reported that an enriched *Staphylococcus* microbiome at 4 weeks of age was associated to physiological immaturity and poorer neurodevelopment at 2 years.^46^ In our study, the main taxa contributing to the microbial signatures for all the development outcomes were *E*. *coli* and *Enterococcus* vs. *Enterobacter*, with *E*. *coli* and *Enterococcus* having a higher relative abundance with respect to *Enterobacter* in the impairment group, and vice versa for the normal group. These findings are supported by previous trials reporting an association between high *Enterococcus* levels in EPT infants during the neonatal period and poor neurodevelopmental outcome at 2 years of age.^47^^,^^49^ Interestingly, the taxa discriminant for the impaired group belong to early maturation stages in the gut microbiota development, while *Enterobacter* is representative of a more mature stage.^42^^,^^68^ Given this observation, our results suggest that a more mature microbiota composition already during the first month of life may have positive consequences on neurodevelopment. This is further supported by the trajectories of the motor outcome, the one with the highest discrimination accuracy (AUC = 0.75), where the impairment group besides having higher relative abundance of *E*. *coli* and *Enterococcus*, those abundances further increased from week 1 to week 4.

In conclusion, this prospective observational study indicates that rather than individual taxa, it is alpha diversity, a balance between certain taxa and, importantly, its maturation over the first month of life that may have an impact on the neurodevelopment at 24 months. Specifically, microbial maturation over the first month was discriminative for motor development, with higher abundance of *E*. *coli*, *Enterococcus*, and *Staphylococcus* relative to *Cutibacterium* and *Enterobacter* being associated with motor impairment. Nonetheless, despite this interesting finding, we acknowledge that the causes of neurodevelopmental impairment are multifactorial and that other factors, such as genetics, gestational age and severe complications, as well as sociodemographic and socioeconomic status, also play important roles. Moreover, our results did not confirm that the previous effects of *L*. *reuteri* DSM 17938 on the neurodevelopment in EPT infants were mediated via the gut microbiome.

### Limitations of the study

A 2-years-of-age follow-up after EPT birth is commonly used for early detection of cerebral palsy, severe cognitive delay, and major sensory impairments. However, an accurate prediction of long-term neurodevelopment may be limited, and an assessment at school age would be more accurate. Another limitation of our study is the substantial proportion of missing data on parental education and maternal language. These factors are relevant as cofactors for language development, particularly because the assessment was conducted in Swedish.

The small and imbalanced sample size between the normal and impaired groups may have limited the statistical power of the study to detect significant differences, potentially explaining the isolated alpha-diversity significant results at 3 weeks of age, although an overall trend toward higher alpha-diversity during the first month of life in the normal group was observed. Another implication of the constrained sample size is the lack of discriminative power of the PLS-DA model and the longitudinal models for language and cognitive outcomes, and consequently the role of the taxa included in signatures should be interpreted carefully.

Future studies should incorporate extended longitudinal follow-up of neurodevelopmental assessments at school age as well as continued microbiome sampling beyond the first month of life, to better characterize long-term neurodevelopmental outcomes and to strengthen causal inference. To better account for socioeconomic and linguistic influences on language development, future cohorts should ensure a complete collection of parental background data. Additionally, larger and more balanced sample sizes will be essential to increase statistical power, reduce group imbalance, and improve the robustness and discriminative capacity of longitudinal models. Well-powered interventions trial studying the effect of factors potentially affecting the gut microbiome in EPT infants, such as unpasteurized donor milk, new probiotics strains, and reduced use of prophylactic antibiotics are warranted.

## Resource availability

### Lead contact

Requests for further information and resources should be directed to and will be fulfilled by the lead contact, Magalí Martí (magali.marti.genero@liu.se).

### Materials availability

This study did not generate new unique reagents.

### Data and code availability

• The 16S rRNA dataset generated in this study is available at the European Nucleotide Archive: PRJEB36531 https://www.ebi.ac.uk/ena/browser/view/PRJEB36531.
• The anonymized coded data have been made available at https://github.com/magge30/PROPEL-2y-neurodevelopment.git
• All original code has been deposited at https://github.com/magge30/PROPEL-2y-neurodevelopment.git and is publicly available.
• Any additional information required to reanalyze the data reported in this study is available from the lead contact upon request.

## Acknowledgments

The computations were partially performed on resources provided by the Swedish National Infrastructure for Computing (SNIC) through 10.13039/501100015701Uppsala Multidisciplinary Center for Advanced Computational Science (10.13039/501100015701UPPMAX) under project SNIC 2020/5-336. We also thank Dr Fredrik Ingemansson, Dr Josefin Lundström, Dr Anders Palm, Dr Björn Westrup and, Dr Laura Österdahl and the study nurses Mrs Christina Fuxin and Mrs Karin Jansmark for their help with the trial; and Dr Stellan Håkansson for help with the SNQ database.

## Author contributions

Conceptualization, T.A., E.S.-E., M.C.J., and M.M.; methodology, M.P., M.L.C., and M.M.; investigation, T.A., E.W., and M.M.; writing – original draft, M.M.; writing – review and editing, T.A., E.W., M.P., M.L.C., E.S-E., M.C.J., and M.M.; funding acquisition, T.A., E.S.-E., M.C.J., and M.M.; resources, T.A., E.S.-E., M.C.J., and M.M.; supervision, T.A., M.C.J., and M.M.

## Declaration of interests

T.A. has received honoraria for lectures and a grant for the present trial from BioGaia AB. M.C.J. has received honoraria for lectures from BioGaia AB. E.S.-E. has received honoraria for lectures and a research grant from BioGaia AB. The other authors declare no competing interests.

## STAR★Methods

### Key resources table

**Table:** 

REAGENT or RESOURCE	SOURCE	IDENTIFIER
**Bacterial and virus strains**		

*Limosilactobacillus reuteri* DSM 17938 for probiotic use	Biogaia AB	*Limosilactobacillus reuteri* DSM 17938
DNA mock control ATCC MSA-2002	https://www.lgcstandards.com/AM/en/search?text=MSA-2002	ATCC-MSA-2002 20 Strain Even Mix Whole Cell Material

**Biological samples**		

Extremely preterm infant stool samples	PROPEL-clinical trial	ClinicalTrials.gov ID: NCT01603368

**Critical commercial assays**		

QIAamp PowerFecal DNA kit (50 preps)	Qiagen	Cat No./ID: 12830-50
16S Metagenomic Sequencing Library Preparation	llumina	Part # 15044223 Rev. B
Nextera XT Index kit v 2 (96 indexes, 384 samples)	llumina	FC-131-2001
Agencourt AMPure XP, 450 mL	Beckman Coulter	A63882
2xKAPA HiFi HotStart ReadyMix	Roche	KK2601
PhiX Control Kit v3	llumina	FC-110-3001
MiSeq Reagent Kit v3 (600-cycle)	llumina	MS-102-3003
EZ1 DNA Tissue Kit	Qiagen	Cat# 953034
SsoFast^TM^ EvaGreen® Supermix	Bio-Rad	Cat# 1725201

**Deposited data**		

Fastq.gz files from 16S rRNA gene sequencing	European Nucleotide Archive (https://www.ebi.ac.uk/ena/browser/home)	Accession number: PRJEB36531
Metadata	https://github.com/magge30/PROPEL-2y-neurodevelopment/blob/main/metadata.csv	Metadata.csv

**Software and algorithms**		

bbduk.sh bbmap/38.08	https://bbmap.org/	BBDuk
FastQC/0.11.5 and MultiQC/1.7	https://www.bioinformatics.babraham.ac.uk/projects/fastqc/https://github.com/ewels/MultiQC	FastQC; MultiQC
DADA2 Pipeline Tuytorial Dada2 version1.10.1	https://benjjneb.github.io/dada2/index.html	N/A
SILVA database version 132	https://www.arb-silva.de/documentation/release-123/	SILVA 132
R Console 4.3.1	https://cran.r-project.org/bin/macosx/	R.4.3.1
Phyloseq R package version 1.46.0	https://joey711.github.io/phyloseq/install.html	N/A
ANCOMBC R package version 2.3.1	https://github.com/FrederickHuangLin/ANCOMBC	N/A
mixOmics R package version 6.24.0	https://mixomics.org/	N/A
SparseMCMM R package version 2.1.1	https://github.com/chanw0/SparseMCMM	N/A
Mediation R package 4.5.0	https://cran.r-project.org/web/packages/mediation/index.html	Version 4.5.0
coda4microbiome R package version 0.1.4	https://malucalle.github.io/coda4microbiome/	N/A
DESeq2 R package 1.42.1	https://github.com/thelovelab/tximport	N/A
mia R package 1.15.6	https://microbiome.github.io/mia/	N/A
IBMSPSS Statistics Software	IBM Corp, Armonk, NY, USA	Version 29

**Other**		

Placebo (Maltodextrin in oil suspension)	BioGaia AB	N/A
Bayley Scales of Infant and Toddler Development, 3rd edition (Bayley-III)	https://doi.org/10.1037/t14978-000	N/A

### Experimental model and study participant details

#### Human preterm infant cohort

This study was part of a prospective randomized, double-blind, placebo-controlled, multi-center trial evaluating the effect of daily supplementation with the probiotic *L*. *reuteri* DSM 17938 in EPT-ELBW infants. The trial was designed to evaluate whether daily *L*. *reuteri* DSM 17938 supplementation improved enteral feeding tolerance. In brief, 134 EPT-ELBW infants (born between gestational week 23 + 0 and 27 + 6, weighing ≤1000 g) were enrolled within the first three days of life and daily supplemented with either *L*. *reuteri* DSM 17938 (1.25 x 10^8^ bacteria (0.2 mL drops) or placebo from birth to post-menstrual week 36. The exclusion criteria were major congenital or chromosomal anomalies, death considered likely within three days after birth, or the infant was included in another intervention trial on growth, feeding intolerance or severe morbidity. The infants were fed exclusively with breast milk (mother’s own milk and/or donor milk) until they had reached a weight of at least 2,000 g. A detailed description of the trial and the clinical outcomes is found in Wejryd et al.^35^

### Method details

#### Two years follow-up

EPT infants undergo a routine follow-up in Sweden according to national guidelines. This includes an assessment with Bayley Scales of Infant and Toddler Development, 3^rd^ edition (Bayley-III).^70^ From the PROPEL-trial, 110 infants participated in the follow up and were assessed by psychologists in Linköping and Stockholm at two years of age. The composite index score for cognition, motor and language scales was used with a Swedish cohort of term born infants used as reference material.^7^ A neonatologist or pediatrician collected information on medical history, physical examination, auxology, standardized neurologic examination, assessment of general appearance of gross- and fine motor function and whether the child was perceived to have normal general development. Data was reported to the https://www.medscinet.com/pnq/and retrieved from the register for the study subjects. The trial was reviewed and approved by the Ethics Committee for Human Research in Linköping (Dnr 2012/28–31, 2016/503-32, 2019–04975). A composite of neurodevelopment impairment was also calculated on data from the follow-up, i.e., Bayley-III, Hammersmith Infant Neurological Examination, diagnosed cerebral palsy, gross motor function classification score, physician’s report on general development, head control, sitting, walking, and fine motor function, parents’ report of speech and walking ability, diagnoses and visual or hearing impairment. If present, the Bayley-III scores were used to define the grade of impairment as follows: normal (score ≥ −2 SD), and impaired (score < −2 SD). This binary classification was chosen because it reflects clinically meaningful cut-offs widely used in neonatal follow-up research and facilitates both interpretation and comparability with prior studies. When Bayley-III results were partially or totally missing, all available data from the following variables at follow-up were used: Hammersmith Infant Neurological Examination, diagnosed cerebral palsy, gross motor function classification score, physician’s report on general development, head control, sitting, walking, and fine motor function, parents’ report of speech and walking ability, diagnoses and impairments of vision or hearing, with the greatest severity recorded used to assign the disability grade. The template for this grading can be found in Table S9.

#### Gut microbiota

16S rRNA gene sequencing was performed on fecal samples collected weekly during the first four weeks (w) of life (1w, 2w, 3w and 4w) as previously described.^31^^,^^71^ Briefly, the V3-V4 hypervariable region of the 16S rRNA gene was sequenced in a paired-end 300 bp sequencing run performed in a MiSeq platform (Illumina). An amplicon sequence variant (ASV) table was generated with the DADA2 Workflow (version 1.10.1).^72^

### Quantification and statistical analysis

Background factors and clinical characteristics were analyzed and compared between groups of infants with normal versus impairment neurodevelopment, using Shapiro-Wilk’s test to assess normality, *t* test for independent samples for continuous data. Categorical data was compared with chi ^2^ – or – if any observed count was five or less, Fisher’s exact test. The background statistics were performed in IBM SPSS Statistics software, version 29 (IBM Corp, Armonk, NY, USA). The microbial causal mediation effect on language development was tested using the Sparse Microbial Causal Mediation Model (SparseMCMM), which is specific for microbiome data, as well as the Casual Mediation Analysis (*mediation* package version 4.5.0) to test for the alpha-diversity mediation.^73^ Alpha-diversity was calculated using Shannon’s diversity index, Pielou’s evenness index, and richness assessed as number of observed ASVs, using the *mia* package (version 1.15.6), and statistically tested for differences between *L*. *reuteri* vs. placebo supplementation groups using *t* test, and between normal vs. impairment neurodevelopment outcomes using logistic regression (*glm()*) as well as generalized linear mixed-effect model (*glmer())* to adjust for confounding variables (Table S2). Microbiome data obtained by high throughput sequencing (16S rRNA or shotgun) represent observed bacterial counts rather than the true absolute abundance of microbes in the environment. As a result, analyses rely on relative abundances, and given the inherent compositional nature of such data, this work employs methods specifically designed to account for compositionality. Inference of differential abundance between the study groups was performed at genus taxonomic level using the Analysis of Compositions of Microbiomes with Bias Correction (ANCOM-BC),^56^ with a prevalence taxa threshold of ≥10%.^74^ This method takes into account the compositionality of microbiome data by modeling bacterial counts with a log-linear regression and applying a bias-correction step, allowing relative abundance data to be interpreted as estimates of underlying absolute abundances. Prior to beta-diversity analyses, variance stabilizing transformation (VST) was applied for normalization across samples,^75^ using the *DESeq2* package.^76^ Bacterial community dissimilarities across the normal and impairment groups and supplementation groups (i.e., beta-diversity) were displayed by non-metric multidimensional scaling (NMDS) plots, and statistically tested using the analysis of similarities (ANOSIM), with 999 permutations as well as Permutational Multivariate Analysis of Variance (adonis2) to control for the confounding variables.^28^^,^^72^ Partial Least Squares – Discriminant Analysis (PLS-DA) was used as a supervised method to model the community at genus and ASV-level for the identification of signatures related to each outcome, using the *mixOmics* package.^77^ The identification of a microbial signature over time that discriminates between the study groups was performed using the *coda4microbiome* R package.^57^ *coda4microbiome* relies on pairwise log-ratios using penalized regression to identify a microbial signature ultimately expressed as a weighted log-contrast (balance) between two groups of taxa, accounting for the compositionality of the data. For that, only individuals with at least three observations across the four weeks (Table 1) and taxa (at genus level) with a prevalence threshold ≥10% were used. Statistical analyses were performed in R version 4.2.1. ∗ denotes *p* < 0.05.

### Additional resources

The study is registered at ClinicalTrials.gov (ID NCT01603368). A detailed protocol for 16S amplicon sequencing characterisation of gut microbiota from extremely preterm infants is available in Martí et STAR protocols.
