# Supplementary material for: Dynamics of early gut microbiota maturation in extremely preterm infants and neurodevelopment at 2 years of age in a probiotic intervention trial
Source: iScience. 2026 Apr 20;29(5):115802. doi: 10.1016/j.isci.2026.115802 (PMC13157099; doi:10.1016/j.isci.2026.115802)
Supplement: Document S1. Figures S1–S3, Tables S1–S6, and S8 [file mmc1.pdf]

iScience, Volume *29*

Supplemental information

Dynamics of early gut microbiota maturation in extremely preterm infants and neurodevelopment at 2 years of age in a probiotic intervention trial

Thomas Abrahamsson, Erik Wejryd, Meritxell Pujolassos, M. Luz Calle, Eva Sverremark-Ekström, Maria C. Jenmalm, and Magalí Martí

This file includes:

.

Table S1: Background and clinical characteristics of preterm infants. Related to Table 2.
Table S2: Gestational age, Site and Multilingual as confounding factors. Related to STAR Methods.
Table S3: Probiotic supplementation in relation to Bayley-II scores. Related to results section *L. reuteri supplementation and neurodevelopment.*
Table S4: Alpha-diversity of ELBW-EPT infants with normal or imparied neurodevelopments outcomes, stratified by Supplementation group. Related to results section *L. reuteri supplementation and neurodevelopment* and Figure 2.
Table S5: Estimated microbial mediation effect. Related to results section *L. reuteri supplementation and neurodevelopment.*
Table S6: Causal Mediation Analysis with alpha-diversity as the mediator. Related to Figure 2.
Table S8: Classification accuracy measures (AUC) for the microbial signatures. Related to Figure 4.
Table S9: Form for assessing neurodevlopmental impairment using Bayley-III. Related to STAR Methods
Figure S1: Alpha-diversity logistic regression modeling. Related to Figure 2.
Figure S2: Non-metric multidimensional scaling (NMDS) of the bacterial community composition. Related to Figure 3.
Figure S3: Microbial signature trajectories. Related to Figure 4.

Table S1. Background and clinical characteristics of the extremely preterm – extremely low birth weight infants from which samples collected at week 1, week 3, week3, and week 4 of life and were compared to Language, Cognition, Motor, and NDI outomes s at 2 years of ages.
t-test for independent samples for continuous data. $\chi 2$-test for categorical data (Fisher's exact test when appropriate). Relatd to Table 2.

Language

	week 1			week 2			week 3			week 4		
**Variables**	**Normal (n=36)**	**Impairment (n=31)**	**p-value[a]**	**Normal (n=38)**	**Impairment (n=29)**	**p value**	**Normal (n=35)**	**Impairment (n=31)**	**p value**	**Normal (n=34)**	**Impairment (n=27)**	**p value**
Gestational age, weeks, mean (SD)	25.7 (1.3)	25.2 (1.3)	0.08	25.8 (1,0)	25.2 (1.4)	**0.03**	25.8 (1)	25.2 (1.3)	0.05	25.7 (1)	25.2 (1.3)	0.10
Birth weight, g, mean (SD)	790 (123)	682 (123)	**0.001**	791 (120)	686 (125)	**0.001**	798 (109)	692 (125)	**0.001**	779 (119)	684 (110)	**0.002**
Birth weight z-score, mean (SD)	-0.8 (1.3)	-1.3 (1.3)	0.10	-0.9 (1.1)	-1.3 (1.3)	0.10	-0.9 (1)	-1.3 (1.3)	0.17	-0.9 (1.1)	-1.3 (1.1)	0.24
Birth length, cm, mean (SD)	33.5 (2.2)	31.8 (2.4)	**0.01**	33.4 (2.3)	32 (2.4)	**0.02**	33.5 (2.2)	32.1 (2.4)	**0.01**	33.3 (2.3)	32.1 (2.3)	**0.04**
Birth length, z-score, mean (SD)	-1,0 (1.7)	-1.7 (1.7)	0.10	-1.2 (1.8)	-1.6 (1.7)	0.19	-1.1 (1.6)	-1.5 (1.6)	0.26	-1.1 (1.7)	-1.5 (1.5)	0.42
Birth head circumference, cm, mean (SD)	23.4 (1.4)	22.5 (1.5)	**<0,001**	23.5 (1.4)	22.6 (1.5)	**0.01**	23,6 (1.4)	22.6 (1.5)	**0.01**	23.5 (1.5)	22.6 (1.4)	**0.01**
Birth head circumference z-score, mean (SD)	-0.7 (0.7)	-0.8 (0.7)	0.40	-0.6 (0.8)	-0.8 (0.6)	0.26	-0.6 (0.8)	-0.8 (0.7)	0.42	-0.6 (0.9)	-0.8 (0.7)	0.29
Apgar at 10 minutes, mean (SD)	8.3 (2)	7.5 (2)	0.07	8.2 (1.7)	7.6 (1.8)	0.10	8.4 (1.4)	7.6 (1.7)	**0.04**	8.2 (1.6)	7.1 (1.9)	**0.02**
SGA, n (%)	28 (78)	23 (74)	0.78	9 (24)	7 (24)	0.97	7 (20)	7 (23)	0.80	9 (26)	6 (22)	0.70
Female sex, n (%)	17 (47)	17 (55)	0.53	18 (47)	15 (52)	0.72	17 (49)	16 (52)	0.81	14 (41)	13 (48)	0.59
Infants from multiple pregnancy, n (%)	14 (39)	8 (26)	0.26	17 (45)	9 (31)	0.25	16 (46)	10 (32)	0.26	15 (44)	9 (33)	0.39
Prenatal steroids administered, n (%)	35 (97)	31 (100)	1.00	37 (97)	29 (100)	1.00	34 (97)	31 (100)	1.00	34 (100)	27 (100)	n.a.
Chorioamnionitis, n (%)	9 (25)	6 (19)	0.58	10 (26)	6 (21)	0.59	9 (26)	7 (23)	0.77	8 (24)	6 (22)	0.90
Cesarean section, n (%)	25 (69)	19 (61)	0.48	27 (71)	18 (62)	0.44	27 (77)	20 (64)	0.26	24 (71)	19 (70)	0.99
Inclusion site	13.5 (11)	15 (8)										
Linkoping, n (%)	10 (28)	18 (58)	**0.02**	11 (29)	17 (59)	**0.01**	10 (29)	17 (55)	**0.03**	9 (26)	16 (59)	**0.01**
Stockholm, n (%)	26 (72)	13 (42)		27 (71)	12 (41)		25 (71)	14 (45)		25 (74)	11 (41)	
**Neonatal hospital care**												
Bronchopulmonary dysplasia, n (%)	19 (53)	24 (77)	**0.04**	20 (53)	22 (76)	0.05	19 (54.3)	24 (77.4)	0.05	18 (53)	23 (85)	**0.01**
Sepsis, n (%)	7 (19)	10 (32)	0.23	9 (24)	9 (31)	0.50	9 (25.7)	10 (32.3)	0.56	9 (26)	5 (19)	0.46
Retinopathy of prematurity, grade 3-5, n (%)	4 (11)	9 (29)	0.06	4 (11)	7 (24)	0.19	4 (11.4)	7 (22.6)	0.19	4 (12)	8 (30)	0.11
Intraventricular haemorrhage, grade 3-4, n (%)	3 (8)	3 (10)	1.00	4 (11)	3 (10)	0.65	4 (11.4)	3 (9.7)	0.57	3 (9)	1 (4)	0.62
Necrotizing enterocolitis, grade 2-3, n (%)	1 (3)	2 (6)	0.44	1 (3)	3 (10)	0.31	0 (0)	4 (12.9)	0.04	2 (6)	3 (11)	0.65
Days to full enteral feeding, median (IQR)	13.5 (12)	15 (8)	0.77	14 (11)	15 (8)	0.97	14 (119	16 (6,5)	0.84	15 (12)	16 (4,5)	0.83
Days on antibiotics week before sampling, median (IQR)	7 (0)	7(0)	0.51	5 (5)	7 (2,75)	0.16	5,5 (7)	7 (7)	0.35	4 (7)	7 (4)	0.10
**Family background**												
Smoking in family at inclusion, n (%)	0 (0)	5 (16)	**0.02**	0 (0)	4 (14)	**0.03**	0 (0)	4 (13)	**0.04**	0 (0)	4 (15)	**0.03**
Parents with postgraduate education												
At least one, n (%)	17 (47)	10 (32)	2x2:0.11	19 (50)	11 (38)	2x2:0.14	16 (46)	11 (35)	2x2:0.12	14 (41)	9 (33)	2x2:0.23
None, n (%)	7 (19)	11 (35)	3x2:0.28	7 (18)	10 (34)	3x2:0.32	6 (17)	11 (35)	3x2:0.24	8 (24)	11 (41)	3x2:0.35
No data, n (%)	12 (33)	10 (32)		12 (32)	8 (28)		13 (36)	9. (29)		12 (35)	7 (26)	
Mother tongue												
Swedish or Scandinavian, n (%)	21 (58)	15 (48)	2x2:0.33	23 (60)	15 (52)	2x2:0.32	21 (60)	16 (52)	2x2:0.34	22 (65)	16 (59)	2x2:0.13
Other, n (%)	5 (14)	8 (26)	3x2:0.46	5 (13)	7 (24)	3x2:0.51	5 (14)	8 (26)	3x2:0.50	2 (6)	6 (22)	3x2:0.15
No data, n (%)	10 (28)	8 (26)		10 (26)	7 (24)		9 (26)	7 (23)		10 (29)	5 (18)	
Multiple languages spoken at home												
No n (%)	27 (75)	18 (58)	2x2:0.51	29 (76)	16 (55)	**2x2:0.03**	28 (80)	16 (52)	**2x2:0.007**	29 (85)	14 (52)	**2x2:0.0001**
Yes, n (%)	7 (19)	13 (42)	3x2:0.07	7 (18)	13 (45)	**3x2:0.04**	5 (14)	15 (48)	**3x2:0.007**	4 (12)	13 (48)	**3x2:0.006**
No data, n (%)	2 (6)	0		2 (5)	0		2 (6)	0		1 (3)	0	

Cognition

Variables	week 1 Normal (n=49)	week 1 Impairment (n=22)	week 1 p value	week 2 Normal (n=48)	week 2 Impairment (n=22)	week 2 p value	week 3 Normal (n=46)	week 3 Impairment (n=23)	week 3 p value	week 4 Normal (n=46)	week 4 Impairment (n=19)	week 4 p value
Gestational age, weeks, mean (SD)	25,6 (1,1)	25,2 (1,5)	0.20	25,7 (1,1)	25,2 (1,5)	0.14	25,7 (1,1)	25,2 (1,4)	0.13	25,6 (1,1)	25,1 (1,4)	0.20
Birth weight, g, mean (SD)	755 (129,7)	686,1 (125,6)	**0.04**	766 (131)	692 (122)	**0.03**	763 (124)	699 (124)	0.05	754 (126)	689 (103)	0.05
Birth weight z-score, mean (SD)	-1 (1,0)	-1,3 (1,5)	0.44	-1,0 (1,1)	-1,2 (1,5)	0.48	-1,0 (1,0)	-1,2 (1,5)	0.54	-1,1 (1,1)	-1,3 (1,4)	0.53
Birth length, cm, mean (SD)	33 (2,5)	31,7 (2,2)	**0.03**	33,1 (2,4)	31,8 (2,2)	**0.03**	33,2 (2,3)	31,9 (2,3)	**0.03**	33,1 (2,4)	31,9 (2,0)	0.07
Birth length, z-score, mean (SD)	-1,2 (1,6)	-1,7 (2)	0.24	-1,2 (1,7)	-1,7 (2)	0.32	-1,2 (1,5)	-1,6 (2,0)	0.33	-1,2 (1,5)	-1,5 (1,8)	0.47
Birth head circumference, cm, mean (SD)	23,1 (1,4)	22,5 (1,4)	0.27	23,2 (1,5)	22,7 (1,4)	0.12	23,2 (1,5)	22,8 (1,5)	0.20	23,2 (1,6)	22,7 (1,4)	0.19
Birth head circumference z-score, mean (SD)	-0,8 (0,8)	-0,7 (0,8)	0.57	-0,7 (0,8)	-0,7 (0,8)	0.82	-0,8 (0,8)	-0,7 (0,8)	0.56	-0,7 (0,8)	-0,7 (0,8)	0.84
Apgar at 10 minutes, mean (SD)	8,2 (1,5)	7,5 (2,2)	0.12	8,1 (1,6)	7,7 (2)	0.38	8,2 (1,4)	7,6 (1,9)	0.20	8 (1,5)	7,0 (2,2)	**0.04**
SGA, n (%)	12 (25)	5 (23)	0.87	12 (25)	5 (23)	0.84	11 (24)	5 (22)	0.84	12 (26)	4 (21)	0.67
Female sex, n (%)	27 (55)	8 (36)	0.14	26 (54)	8 (36)	0.17	27 (59)	8 (35)	0.06	23 (50)	5 (26)	0.08
Infants from multiple pregnancy, n (%)	16 (33)	8 (36)	0.76	19 (40)	9 (41)	0.92	18 (39)	10 (44)	0.73	17 (37)	9 (47)	0.44
Prenatal steroids administered, n (%)	48 (98)	22 (100)	1.00	47 (98)	22 (100)	1.00	45 (98)	23(100)	1.00	46 (100)	19 (100)	n.a.
Chorioamnionitis, n (%)	11 (22)	4 (18)	0.76	11 (23)	5 (23)	0.99	12 (26)	5 (22)	0.69	10 (22)	4 (21)	1.00
Cesarean section, n (%)	32 (65)	14 (64)	0.89	33 (69)	14 (64)	0.67	34 (74)	15 (65)	0.45	31 (67)	14 (74)	0.62
Inclusion site												
Linkoping, n (%)	18 (37)	11 (50)	0.29	19 (40)	10 (46)	0.64	18 (39)	10 (44)	0.73	17 (37)	9 (47)	0.44
Stockholm, n (%)	31 (63)	11 (50)		29 (60)	12 (55)		28 (61)	13 (56)		29 (63)	10 (53)	
**Neonatal hospital care**												
Bronchopulmonary dysplasia, n (%)	28 (57)	17 (77)	0.10	27 (56)	17 (77)	0.09	26 (56)	18 (78)	0.08	27 (69)	17 (89)	**0.02**
Sepsis, n (%)	10 (20)	8 (36)	0.15	11 (23)	8 (36)	0.24	11 (24)	9 (39)	0.19	11 (24)	4 (21)	0.80
Retinopathy of prematurity, grade 3-5, n (%)	7 (14)	6 (27)	0.20	6 (13)	5 (23)	0.30	6 (13)	5 (22)	0.49	6 (13)	6 (32)	0.16
Intraventricular haemorrhage, grade 3-4, n (%)	3 (6)	3 (14)	0.37	4 (8)	3 (14)	0.67	4 (9)	3 (13)	0.68	3 (7)	1 (5)	1.00
Necrotizing enterocolitis, grade 2-3, n (%)	1 (2)	2 (9)	0.23	1 (2)	3 (14)	0.09	0 (0)	4 (17)	0.01	2 (4)	3 (16)	0.14
Days to full enteral feeding, median (IQR)	15 (10)	13.5 (8)	0.77	15,5 (9,5)	14 (11)	0.88	16 (9)	14 (12,5)	0.95	16 (11)	14 (9)	0.94
Days on antibiotics week before sampling, median (IQR)	7 (0)	7(0)	0.62	6 (5)	7 (3)	0.98	7 (7)	7 (7)	0.90	4 (7)	7 (5,5)	0.37
**Family background**												
Smoking in family at inclusion, n (%)	4 (8)	2 (9)	1.00	3 (6)	2 (9)	0.65	3 (6)	2 (9)	1.00	3 (7)	2 (10)	0.63
Parents with postgraduate education												
At least one, n (%)	18 (37)	9 (41)	2x2:0.77	20 (42)	10 (45)	2x2:0.90	17 (37)	10 (43)	2x2:0.84	15 (33)	8 (42)	2x2:0.91
None, n (%)	13 (27)	8 (36)	3x2:0.48	13 (27)	7 (32)	3x2:0.76	12 (26)	8 (35)	3x2:0.43	14 (30)	8 (42)	3x2:0.24
No data, n (%)	18 (37)	5 (23)		15 (31)	5 (23)		17 (37)	5 (22)		17 (37)	3 (16)	
Mother tongue												
Swedish or Scandinavian, n (%)	27 (55)	12 (54)	2x2:0.75	29 (60)	12 (55)	2x2:0.49	27 (59)	13 (56)	2x2:0.74	28 (61)	13 (68)	2x2:1.00
Other, n (%)	9 (18)	5 (23)	3x2:0.89	7 (15)	5 (23)	3x2:0.70	8 (17)	5 (22)	3x2:0.91	6 (13)	3 (16)	3x2:0.67
No data, n (%)	13 (26)	5 (23)		12 (25)	5 (23)		11 (24)	5 (22)		12 (26)	3 (16)	
Multiple languages spoken at home												
No n (%)	33 (67)	15 (68)	2x2:0.86	34 (70)	14 (64)	2x2:0.38	33 (72)	14 (61)	2x2:0.23	35 (76)	12 (63)	2x2:0.23
Yes, n (%)	14 (29)	7 (32)	3x2:0.61	12 (25)	8 (36)	3x2:0.43	11 (24)	9 (39)	3x2:0.29	10 (22)	7 (37)	3x2:0.39
No data, n (%)	2 (4)	0		2 (4)	0		2 (4)	0		1 (2)	0	

Motor

Variables	week 1 Normal (n=49)	week 1 Impairment (n=13)	week 1 p value	week 2 Normal (n=49)	week 2 Impairment (n=14)	week 2 p value	week 3 Normal (n=46)	week 3 Impairment (n=15)	week 3 p value	week 4 Normal (n=45)	week 4 Impairment (n=13)	week 4 p value
Gestational age, weeks, mean (SD)	25,6 (1,2)	24,8 (1,2)	**0.02**	25,6 (1,2)	24,9 (1,2)	0.06	25,7 (1,1)	25,0 (1,2)	0.06	25,6 (1,1)	24,8 (1,0)	**0.02**
Birth weight, g, mean (SD)	755 (128)	687 (139)	0.10	758 (131)	706 (133)	0.20	757 (126)	716 (134)	0.28	751 (127)	712 (118)	0.32
Birth weight z-score, mean (SD)	-1,1 (1,2)	-0,9 (1,4)	0.70	-1,1 (1,2)	-0,9 (1,3)	0.65	-1,1 (1,1)	-0,9 (1,3)	0.58	-1,2 (1,2)	-0,7 (0,8)	0.21
Birth length, cm, mean (SD)	33,1 (2,5)	31,9 (2,4)	0.14	33,1 (2,4)	31,9 (2,3)	0.10	33,2 (2,3)	32,1 (2,3)	0.12	22,1 (2,4)	32,4 (2,0)	0.36
Birth length, z-score, mean (SD)	-1,2 (1,6)	-1,1 (2)	0.75	-1,2 (1,6)	-1,3 (2,2)	0.88	-1,1 (1,5)	-1,2 (2,1)	0.91	-1,3 (1,6)	-0,7 (1,2)	0.25
Birth head circumference, cm, mean (SD)	23,1 (1,5)	22,6 (1,3)	0.32	23,2 (1,5)	22,8 (1,3)	0.35	23,2 (1,5)	22,9 (1,4)	0.49	23,3 (1,6)	22,8 (1,4)	0.31
Birth head circumference z-score, mean (SD)	-0,8 (0,8)	-0,4 (0,5)	0.08	-0,7 (0,8)	-0,4 (0,5)	0.17	-0,7 (0,8)	-0,4 (0,5)	0.10	-0,7 (0,8)	-0,3 (0,4)	**0.02**
Apgar at 10 minutes, mean (SD)	8,3 (1,5)	7,1 (2,4)	0.10	8,2 (1,6)	7,4 (2,0)	0.11	8,4 (1,4)	7,3 (1,9)	**0.03**	8,2 (1,5)	6,5 (2,0)	**0.003**
SGA, n (%)	14 (29)	1 (8)	0.12	14 (29)	1 (7)	0.16	13 (28)	1 (7)	0.16	14 (31)	0 (0)	**0.03**
Female sex, n (%)	26 (53)	3 (23)	0.07	25 (51)	4 (29)	0.14	25 (54)	4 (27)	0.06	22 (49)	1 (8)	**0.01**
Infants from multiple pregnancy, n (%)	17 (35)	4 (31)	1.00	19 (39)	6 (43)	0.78	18 (39)	7 (47)	0.61	18 (40)	5 (38)	0.92
Prenatal steroids administered, n (%)	48 (98)	13 (100)	1.00	48 (98)	14 (100)	1.00	45 (98)	15 (100)	1.00	45 (100)	13 (100)	n.a.
Chorioamnionitis, n (%)	10 (20)	2 (15)	1.00	10 (20)	4 (29)	0.52	10 (22)	4 (27)	0.69	9 (20)	2 (15)	1.00
Cesarean section, n (%)	32 (65)	38 (61)	0.21	33 (67)	7 (50)	0.23	33 (72)	8 (53)	0.22	31 (69)	7 (54)	0.34
Inclusion site												
Linkoping, n (%)	17 (35)	10 (77)	**0.01**	18 (37)	9 (64)	0.07	17 (37)	9 (60)	0.12	16 (36)	8 (62)	0.09
Stockholm, n (%)	32 (65)	3 (23)		31 (63)	5 (36)		29 (63)	6 (40)		29 (64)	5 (38)	
**Neonatal hospital care**												
Bronchopulmonary dysplasia, n (%)	27 (55)	11 (85)	0.05	27 (55)	11 (79)	0.11	25 (54)	12 (80)	0.13	25 (56)	12 (92)	**0.02**
Sepsis, n (%)	10 (20)	4 (31)	0.47	10 (20)	5 (36)	0.29	10 (22)	6 (40)	0.19	10 (22)	2 (15)	0.72
Retinopathy of prematurity, grade 3-5, n (%)	6 (12)	4 (31)	0.20	5 (10)	3 (21)	0.36	5 (11)	3 (20)	0.39	5 (11)	4 (31)	0.10
Intraventricular haemorrhage, grade 3-4, n (%)	3 (6)	2 (15)	0.28	3 (6)	3 (21)	0.12	3 (6)	3 (20)	0.15	3 (7)	1 (8)	1.00
Necrotizing enterocolitis, grade 2-3, n (%)	2 (4)	1 (8)	0.51	2 (4)	2 (14)	0.21	1 (2)	3 (20)	**0.04**	3 (7)	2 (15)	0.31
Days to full enteral feeding, median (IQR)	15 (9)	14 (8)	0.59	15 (10)	15 (11)	0.35	15,5 (9)	16 (14,5)	0.24	16 (10)	14(8)	0.79
Days on antibiotics week before sampling, median (IQR)	7 (0)	7(0)	0.36	6,5 (5)	7 (3)	0.30	7 (7)	7 (7)	0.79	4,5 (7)	3,5 (7)	0.82
**Family background**												
Smoking in family at inclusion, n (%)	3 (6)	2 (15)	0.28	2 (4)	2 (14)	0.17	2 (4)	2 (13)	0.25	2 (4)	2 (15)	0.21
Parents with postgraduate education												
At least one, n (%)	20 (41)	6 (46)		21 (43)	8 (57)		18 (39)	8 (53)		17 (38)	5 (38)	
None, n (%)	12 (24)	7 (54)	2x2:0.31<br>3x2:0.07	12 (24)	6 (43)	2x2:0.67<br>3x2:0.12	11 (24)	7 (47)	**2x2:0.58**<br>**3x2:0.05**	13 (29)	7 (54)	2x2:0.50<br>3x2:0.12
No data, n (%)	17 (35)	0 (0)		16 (33)	0 (0)		17 (37)	0 (0)		15 (33)	1 (8)	
Mother tongue												
Swedish or Scandinavian, n (%)	28 (57)	9 (69)		30 (61)	9 (64)		28 (61)	10 (67)		29 (64)	10 (77)	
Other, n (%)	7 (14)	4 (31)	2x2:0.46<br>3x2:0.06	6 (12)	5 (36)	**2x2:0.14**<br>**3x2:0.03**	6 (13)	3 (33)	**2x2:0.69**<br>**3x2:0.04**	4 (9)	3 (23)	2x2:0.38<br>3x2:0.07
No data, n (%)	14 (29)	0 (0)		13 (26)	0 (0)		12 (26)	0 (0)		12 (27)	0 (0)	
Multiple languages spoken at home												
No n (%)	35 (71)	7 (54)		35 (71)	7 (50)		34 (74)	7 (47)		35 (78)	7 (54)	
Yes, n (%)	12 (24)	6 (46)	2x2:0.15<br>3x2:0.26	12 (24)	7 (50)	**2x2:0.08**<br>**3x2:0.02**	10 (22)	8 (53)	**2x2:0.05**<br>**3x2:0.06**	9 (20)	6 (46)	2x2:0.06<br>3x2:0.15
No data, n (%)	2 (4)	0 (0)		2 (4)	0 (0)		2 (4)	0 (0)		1 (2)	0 (0)	

NDI

Variables	week 1 Normal (n=48)	week 1 Impairment (n=42)	week 1 p value	week 2 Normal (n=50)	week 2 Impairment (n=42)	week 2 p value	week 3 Normal (n=43)	week 3 Impairment (n=45)	week 3 p value	week 4 Normal (n=44)	week 4 Impairment (n=41)	week 4 p value
Gestational age, weeks, mean (SD)	25,8 (1,0)	25,0 (1,3)	**<0,001**	25,9 (1,0)	25,0 (1,3)	**<0.001**	26,0 (0,9)	25,0 (1,3)	**<0,001**	25,9 (1,0)	25,0 (1,2)	**<0,001**
Birth weight, g, mean (SD)	796 (126)	682 (120)	**<0,001**	799 (128)	686 (121)	**<0.001**	807 (114)	688 (121)	**<0,001**	799 (125)	695 (117)	**<0,001**
Birth weight z-score, mean (SD)	-0.9 (1,1)	-1,1 (1,3)	0.21	-0,9 (1,1)	-1,1 (1,2)	0.27	-0,9 (1,0)	-1,1 (1,3)	0.45	-1,0 (1,1)	-1,0 (1,1)	0.86
Birth length, cm, mean (SD)	33,6 (2,5)	31,6 (2,1)	**<0,001**	33,6 (2,5)	31,6 (2,1)	**<0,001**	33,9 (2,2)	31,7 (2,1)	**<0,001**	33,6 (2,5)	31,9 (2,0)	**<0,001**
Birth length, z-score, mean (SD)	-1,1 (1,8)	-1,6 (1,6)	0.07	-1,1 (1,8)	-1,6 (1,6)	0.09	-1,0 (1,5)	-1,6 (1,6)	0.06	-1,1 (1,8)	-1,4 (1,4)	0.44
Birth head circumference, cm, mean (SD)	23,3 (1,3)	22,4 (1,4)	**0.001**	23,4 (1,4)	22,5 (1,5)	**<0,001**	25,6 (1,3)	22,5 (1,5)	**<0,001**	23,5 (1,4)	22,5 (1,4)	**0.001**
Birth head circumference z-score, mean (SD)	-0,8 (0,7)	-0,7 (0,7)	0.28	-0,8 (0,8)	-0,7 (0,7)	0.27	-0,8 (0,7)	-0,7 (0,7)	0.55	-0,8 (0,8)	-0,7 (0,7)	0.66
Apgar at 10 minutes, mean (SD)	8,2 (1,7)	7,6 (1,8)	0.08	8,1 (1,8)	7,6 (1,7)	0.10	8,4 (1,4)	7,6 (1,7)	**0.02**	8,1 (1,8)	7,3 (1,8)	**0.03**
SGA, n (%)	12 (25)	8 (19)	0.50	13 (26)	7 (17)	0.28	11 (26)	8 (18)	0.44	13 (30)	6 (15)	0.10
Female sex, n (%)	25 (52)	17 (40)	0.27	26 (52)	17 (40)	0.27	24 (56)	18 (40)	0.14	23 (52)	14 (34)	0.09
Infants from multiple pregnancy, n (%)	18 (38)	12 (29)	0.37	21 (42)	15 (36)	0.67	19 (44)	16 (36)	0.41	20 (46)	13 (32)	0.19
Prenatal steroids administered, n (%)	46 (96)	42 (100)	0.50	48 (96)	42 (100)	0.50	42 (98)	45 (100)	0.49	43 (98)	41 (100)	1.00
Chorioamnionitis, n (%)	14 (29)	6 (14)	0.09	15 (30)	8 (19)	0.23	13 (30)	9 (20)	0.27	13 (30)	7 (17)	0.18
Cesarean section, n (%)	33 (69)	25 (60)	0.32	35 (70)	25 (60)	0.29	33 (77)	29 (64)	0.21	33 (75)	26 (63)	0.25
Inclusion site												
Linkoping, n (%)	10 (21)	22 (54)	**0.002**	11 (22)	22 (52)	**0.002**	10 (23)	22 (49)	**0.01**	9 (20)	21 (51)	**0.004**
Stockholm, n (%)	38 (79)	20 (48)		39 (78)	28 (48)		33 (77)	23 (51)		35 (80)	20 (49)	
**Neonatal hospital care**												
Bronchopulmonary dysplasia, n (%)	20 (42)	32 (76)	**<0,001**	21 (42)	32 (76)	**<0,001**	17 (40)	35 (78)	**<0,001**	18 (50)	34 (83)	**<0,001**
Sepsis, n (%)	11 (23)	14 (33)	0.27	12 (24)	13 (31)	0.46	10 (23)	16 (36)	0.21	11 (25)	9 (22)	0.74
Retinopathy of prematurity, grade 3-5, n (%)	3 (6)	12 (29)	**0.01**	3 (6)	9 (21)	**0.03**	3 (7)	10 (22)	**0.04**	3 (7)	11 (27)	0.01
Intraventricular haemorrhage, grade 3-4, n (%)	3 (6)	6 (14)	0.30	3 (6)	8 (19)	0.06	3 (7)	8 (18)	0.13	3 (7)	5 (12)	0.47
Necrotizing enterocolitis, grade 2-3, n (%)	1 (2)	3 (7)	0.34	1 (2)	4 (10)	0.17	0 (0)	5 (11)	0.06	2 (4)	4 (10)	0.42
Days to full enteral feeding, median (IQR)	14 (10)	14,5 (10)	0.32	14.5 (10)	15 (9)	0.44	15 (10)	16 (8)	0.41	15 (11)	15(7)	0.38
Days on antibiotics week before sampling, median (IQR)	7 (0)	7(0)	0.84	4,5 (5)	7 (3)	0.12	7 (7)	7 (6,75)	0.25	5 (7)	4 (7)	0.09
**Family background**												
Smoking in family at inclusion, n (%)	1 (2)	6 (14)	0.05	1 (2)	5 (12)	0.09	0 (0)	5 (11)	0.06	1 (2)	6 (15)	0.05
Parents with postgraduate education												
At least one, n (%)	16 (33)	13(31)	**2x2:0.07**	17 (34)	16 (38)	**2x2:0.15**	14 (33)	16 (36)	**2x2:0.15**	12 (27)	13 (31)	**2x2:0.30**
None, n (%)	7 (15)	16 (38)	**3x2:0.03**	7 (14)	15 (36)	**3x2:0.02**	6 (14)	16 (36)	**3x2:0.03**	8 (18)	16 (39)	**3x2:0.04**
No data, n (%)	25 (52)	13(31)		26 (52)	11 (26)		23 (53)	13 (29)		24 (55)	12 (29)	
Mother tongue												
Swedish or Scandinavian, n (%)	30 (62)	22 (52)	2x2:0.06	33 (66)	23 (55)	**2x2:0.00**	28 (65)	25 (56)	**2x2:0.03**	29 (66)	25 (61)	**2x2:0.04**
Other, n (%)	6 (12)	13(31)	3x2:0.09	5 (10)	12 (29)	**3x2:0.07**	4 (9)	14 (31)	**3x2:0.03**	3 (7)	11 (27)	**3x2:0.02**
No data, n (%)	12 (25)	7 (17)		12 (24)	7 (17)		11 (26)	6 (13)		12 (27)	5 (12)	
Multiple languages spoken at home												
No n (%)	32 (67)	28 (67)	2x2:0.43	33 (66)	28 (67)	**2x2:0.48**	30 (70)	28 (62)	2x2:0.13	32 (73)	27 (66)	2x2:0.10
Yes, n (%)	11 (23)	14 (33)	3x2:0.07	11 (22)	14 (33)	**3x2:0.05**	8 (19)	16 (36)	3x2:0.07	7 (16)	13 (32)	3x2:0.09
No data, n (%)	5 (10)	0 (0)		6 (12)	0 (0)		5 (12)	1 (2)		5 (11)	1 (2)	

Table S2. Gestational age, Site and Multilingual as confounding factors. Related to STAR Methods. Grey cells indicate cases where these variables were only significantly associated with neurodevelopmental outcomes. Black cells indicate cases where these variables were adjusted for, as they were also significantly associated with microbiome measures.

Microbiome assessment	Neurodevelopment outcome	Gestational age				Site				Multilingual			
		w1	w2	w3	w4	w1	w2	w3	w4	w1	w2	w3	w4
Differential abundance	Language		Grey			Black	Grey	Grey	Grey		Grey	Grey	Grey
	Motor	Grey			Grey	Black							
	NDI	Grey	Black	Grey	Black	Black	Grey	Black	Black				
Alpha diversity	Language		Grey			Black	Grey	Grey	Grey		Grey	Grey	Grey
	Motor	Grey			Grey	Black							
	NDI	Grey	Grey	Grey	Grey	Black	Black	Grey	Grey				
Beta diversity	Language		Grey			Black	Grey	Grey	Grey		Grey	Grey	Grey
	Motor	Black			Grey	Black							
	NDI	Black	Grey	Grey	Grey	Black	Grey	Grey	Grey				

Table S3. Probiotic supplementation in relation to Bayley-III scores (chi-square test), Alpha-diversity (T-test and FDR correction), Beta-diversity (ANOSIM test), and differential abundance (ANCOM test). Related to results section *L. reuteri supplementation and neurodevelopment.*
SD: standard deviation; lfc: log fold change placebo vs *L. reuteri.*

Outcome	Supplementation	Timepoint	N	Neurodevelopment			Alpha-diversity						Beta-diversity		ANCOM	
				Impairment	Normal	Chi2 p-value	Shannon q-value	Shannon N(SD)	Richness q-value	Richness N(SD)	Evenness q-value	Evenness N(SD)	Anosim significance	Anosim Statistic R	Genus	lfc
Language	Placebo	w1	36	17	19	1	**0.00**	1(0.4)	0.15	12.3(6.3)	**0.001**	0.4(0.2)	0.7	0.0	*Lactobacillus*	6.8
	L. reuteri		31	14	17			1.7(0.4)		16.9(5.9)		0.6(0.1)				
	Placebo	w2	36	16	20	1	**0.00**	1.1(0.5)	0.40	19.2(6.5)	**0.01**	0.4(0.2)	0.9	0.0	*Lactobacillus*	5.9
	L. reuteri		31	13	18			1.6(0.4)		23.4(5.9)		0.5(0.1)				
	Placebo	w3	35	17	18	0.98	0.10	1.1(0.6)	0.37	19.9(7.3)	0.23	0.4(0.2)	0.8	0.0	*Lactobacillus*	4.9
	L. reuteri		31	14	17			1.5(0.5)		24.9(7.2)		0.5(0.1)				
	Placebo	w4	30	14	16	0.91	1.00	1.3(0.5)	1.00	21.9(7.2)	1.00	0.4(0.2)	0.8	0.0	*Lactobacillus*	3.5
	L. reuteri		31	13	18			1.6(0.5)		24.8(6.2)		0.5(0.1)				
Cognition	Placebo	w1	40	12	28	1	**0.00**	1.0(0.4)	**0.03**	11.8(6.3)	**0.00**	0.4(0.2)	1.0	-0.1	*Lactobacillus*	7.0
	L. reuteri		31	10	21			1.7(0.4)		16.9(5.9)		0.6(0.1)				
	Placebo	w2	39	12	27	1	**0.00**	1.1(0.5)	1.00	20.1(7.9)	**0.02**	0.4(0.2)	0.4	0.0	*Lactobacillus*	5.8
	L. reuteri		31	10	21			1.6(0.4)		23.4(5.9)		0.5(0.1)				
	Placebo	w3	38	13	25	1	0.24	1.1(0.6)	0.74	20.4(7.7)	0.45	0.4(0.2)	0.7	0.0	*Lactobacillus*	4.9
	L. reuteri		31	10	21			1.5(0.5)		24.9(7.2)		0.5(0.1)				
	Placebo	w4	33	10	23	1	1.00	1.4(0.6)	1.00	23.0(8)	1.00	0.4(0.2)	0.3	0.0	*Lactobacillus*	3.6
	L. reuteri		32	9	23			1.6(0.5)		25.0(6.2)		0.5(0.1)				
Motor	Placebo	w1	37	8	29	1	**0.00**	1.0(0.4)	0.09	12.1(6.4)	**0.00**	0.4(0.2)	1.0	-0.2	*Lactobacillus*	6.7
	L. reuteri		25	5	20			1.7(0.4)		17.4(6.1)		0.6(0.1)				
	Placebo	w2	37	8	29	1	**0.02**	1.1(0.5)	1.00	19.6(7.5)	0.10	0.4(0.2)	0.8	-0.1	*Lactobacillus*	6.0
	L. reuteri		26	6	20			1.6(0.5)		23.0(6.2)		0.5(0.1)				
	Placebo	w3	36	9	27	1	0.09	1.1(0.6)	0.15	20.1(7.2)	0.31	0.4(0.2)	1.0	-0.2	*Lactobacillus*	5.0
	L. reuteri		25	6	19			1.6(0.5)		26.0(7.4)		0.5(0.2)				
	Placebo	w4	31	7	24	1	1.00	1.4(0.5)	1.00	22.8(6.9)	1.00	0.4(0.2)	0.4	0.0	*Lactobacillus*	3.5
	L. reuteri		27	6	21			1.6(0.5)		25.2(6.5)		0.5(0.1)			*Finegoldia*	-5.7
NDI	Placebo	w1	47	22	25	1	**0.000**	1.0(0.4)	**0.002**	12.2(6.2)	**0.000**	0.4(0.2)	0.1	0.0	*Lactobacillus*	6.8
	L. reuteri		43	20	23			1.7(0.4)		17.6(5.6)		0.6(0.1)				
	Placebo	w2	48	22	26	1	**0.000**	1.1(0.5)	0.130	20.2(7.4)	**0.000**	0.4(0.2)	0.5	0.0	*Lactobacillus*	6.3
	L. reuteri		44	20	24			1.7(0.4)		25.1(7.8)		0.5(0.1)				
	Placebo	w3	45	23	22	1	0.560	1.2(0.6)	0.160	20.1(7.3)	1.000	0.4(0.2)	0.2	0.0	*Lactobacillus*	3.9
	L. reuteri		43	22	21			1.5(0.5)		24.8(7.2)		0.5(0.2)				
	Placebo	w4	41	21	20	0.753	1.000	1.3(0.5)	1.000	22.7(7.7)	1.000	0.4(0.2)	0.9	0.0	*Lactobacillus*	4.1
	L. reuteri		44	20	24			1.6(0.5)		24.2(7.6)		0.5(0.2)				

Table S4. Gut microbiota alpha-diversity of ELBW-EPT infants with normal or imparied neurodevelopments outcomes, stratified by Supplementation group. Related to results section *L. reuteri supplementation and neurodevelopment* and Figure 2.
T-test with FDR (q-value)

		**Language**		**Cognition**		**Motor**		**NDI**	
	Alpha-diversity	**Placebo**	**Placebo**	**Placebo**	**Placebo**	**Placebo**	**Placebo**	**Placebo**	**Placebo**
**w1**	Diversity	0.28	0.74	0.61	0.73	0.88	0.89	0.21	0.85
	Richness	0.33	0.58	0.14	0.43	0.06	0.10	0.10	0.30
	Evenness	0.24	0.55	0.69	0.87	0.63	0.10	0.45	0.57
**w2**	Diversity	0.11	0.95	0.57	0.64	0.36	0.81	0.76	0.24
	Richness	0.10	0.76	0.61	0.63	0.19	0.40	0.29	0.03
	Evenness	0.16	0.91	0.57	0.45	0.65	0.49	0.87	0.74
**w3**	Diversity	**0.01 (q=0.08)**	0.79	0.34	0.84	0.12	0.17	**0.004 (q = 0.02)**	0.88
	Richness	**0.026 (q=0.10)**	0.45	0.66	0.95	0.09	1.00	0.16	0.18
	Evenness	**0.013 (q=0.08)**	0.99	0.28	0.82	0.18	0.12	**0.002 (q = 0.02)**	0.52
**w4**	Diversity	0.23	0.41	0.93	0.59	0.12	0.87	0.16	0.94
	Richness	0.32	0.63	0.50	0.73	0.81	0.20	0.35	0.16
	Evenness	0.23	0.31	0.85	0.54	**0.048 (q=0.29)**	0.81	0.17	0.34

Table S5. Estimated microbial mediation effect (SparseMCMM). Related to results section *L. reuteri supplementation and neurodevelopment.*

		Estimated P-value		Estimated causal effects		
**Outcome**	**Time point**	**OME**	**CME**	**ME**	**DE**	**TE**
Language	w1	0.396	0.416	-66.018	71.578	5.561
	w2	0.446	0.089	14.917	-2.670	12.247
	w3	0.386	0.446	-62.164	146.885	84.721
	w4	0.366	0.347	52.115	-53.591	-1.475
Cognition	w1	0.396	0.416	32.054	-32.441	-0.387
	w2	0.475	0.317	-12.057	11.611	-0.446
	w3	0.455	0.485	30.182	-16.671	13.511
	w4	0.426	0.406	-42.678	41.848	-0.830
Motor	w1	0.356	0.376	34.225	-35.543	-1.318
	w2	0.545	0.465	-8.807	19.385	10.579
	w3	0.554	0.545	35.447	-21.397	14.050
	w4	0.782	0.604	3.773	15.194	18.967

OME: tests the overall mediation effect of the microbiome community
CME: test whether at least one taxon has a mediation effect.
ME: estimates of the overall microbial mediation effect
DE: estimates of the direct treatment effect.
TE: estimates of the total treatment effect.

Table S6. Causal Mediation Analysis with alpha-diversity as the mediator. Related to Figure 2.

		week 1				week 2				week 3				week 4			
		**Estimate**	**95% CI Lower**	**95% CI Upper**	**p-value**	**Estimate**	**95% CI Lower**	**95% CI Upper**	**p-value**	**Estimate**	**95% CI Lower**	**95% CI Upper**	**p-value**	**Estimate**	**95% CI Lower**	**95% CI Upper**	**p-value**
**Diversity**	Total Effect	7.13	0.23	14.94	0.05	7.26	0.21	15.46	0.04	8.08	0.66	15.32	0.03	6.75	-0.94	14.67	0.09
	ACME	1.42	-4.79	10.78	0.64	1.63	-2.62	8.68	0.42	0.82	-2.65	4.39	0.75	0.34	-2.31	3.86	0.87
	ADE	5.70	-4.19	14.44	0.23	5.64	-2.51	13.97	0.18	7.26	-0.66	15.67	0.07	6.41	-1.14	14.33	0.10
	Prop. Mediated	0.20	-1.52	2.33	0.64	0.22	-0.71	1.77	0.45	0.10	-0.56	0.99	0.76	0.05	-0.91	0.92	0.86
**Richness**	Total Effect	7.23	0.26	14.47	0.04	7.26	0.21	15.46	0.04	7.35	0.61	15.25	0.03	6.34	-0.61	14.36	0.07
	ACME	1.78	-0.72	5.67	0.18	1.63	-2.62	8.68	0.42	1.32	-0.60	4.53	0.15	0.38	-1.45	3.15	0.51
	ADE	5.46	-1.45	12.71	0.13	5.64	-2.51	13.97	0.18	6.03	-1.68	14.60	0.13	5.96	-1.31	14.01	0.12
	Prop. Mediated	0.25	-0.24	1.32	0.20	0.22	-0.71	1.77	0.45	0.18	-0.12	1.63	0.18	0.06	-0.46	1.07	0.54
**Evenness**	Total Effect	7.08	-0.15	16.61	0.06	6.92	-0.22	16.65	0.06	7.63	0.32	15.70	0.04	6.68	-1.65	15.60	0.11
	ACME	0.75	-4.17	9.95	0.68	0.36	-3.44	9.30	0.71	-0.03	-3.43	4.68	0.96	0.11	-3.53	4.85	0.97
	ADE	6.33	-2.35	14.63	0.14	6.57	-1.18	14.70	0.09	7.66	-0.09	15.91	0.05	6.57	-0.96	14.49	0.09
	Prop. Mediated	0.11	-1.42	1.69	0.68	0.05	-0.91	1.18	0.70	0.00	-0.83	0.79	0.96	0.02	-1.48	1.13	0.92

Total Effect: indicates whether probiotic supplementation affects Language development.
ACME: average Causal Mediation Effect (indirect effect), indicates whether alpha-diversity had a mediating effect.
ADE: Average Direct Effect.

Table S8. Classification accuracy measures. Apparent AUC (AUC of the signature applied to the same data that was used to generate the model), mean cross-validation (cv) AUC, standard deviation (sd) cv-AUC. Related to Figure 4.

	**apparent AUC**	**mean cv-AUC**	**sd cv-AUC**
Language	0.65	0.55	0.04
Cognition	0.63	0.49	0.10
Motor	0.80	0.74	0.02
NDI	0.63	0.58	0.05

Table S9. Form for assessing neurodevlopmental impairment (NDI) using Bayley-III and/or clinical data. Related to STAR Methods.

**Developmental domain**	**Classification of neuroimpairment in each developmental domain**			
	**None (0)**	**Mild (1)**	**Moderate (2)**	**Severe (3)**
**Cognition OR language**				
Bayley III cognitive scales	Bayley III<br>Scores > -1 SD<br>(Index ≥ 95)	Bayley III<br>Scores -1 SD to -2 SD<br>(Index 83-94)	Bayley III<br>Scores -2 SD to -3 SD<br>(Index 72-82)	Bayley III<br>Scores < -3 SD<br>(Index <72)
Bayley III language scales	Bayley III<br>Scores > -1 SD<br>(Index ≥ 97)	Bayley III<br>Scores -1 SD to -2 SD<br>(Index 85-96	Bayley III<br>Scores -2 SD to -3 SD<br>(Index 72-84)	Bayley III<br>Scores < -3 SD<br>(Index <72)
Parent report	Speech:<br>Uses sentences with 2-3 words	Speech:<br>Says a few words (vocabulary > 10 words)	Speech:<br>Says a few words (vocabulary < 10 words)	Speech:<br>Does not speak at all
**OR Motor function**				
CP (GMFCSF)	None	1	2-3	4-5 (non ambulant)
Bayley III fine and gross motor scales	> -1 SD<br>(Index ≥ 94)	Scores -1 SD to -2 SD<br>(Index 80-93)	Scores -2 SD to -3 SD<br>(Index 66-79)	Scores < -3 SD<br>(Index <66)
Doctor´s report	Normal head control<br>Sits without support<br>Walks without support<br><br>Normal finger movements	Fine motor function:<br>Clumsy but can grasp<br>OR<br>Abnormal neurology or motor development	Fine motor function:<br>Clumsy but can grasp	Unsteady head control<br>Unsteady sitting or cannot sit.<br>Fine motor: Does not grasp objects.
**OR Hearing**				
Parent report	No hearing impairment	No hearing impairment	Hearing impairment<br>OR<br>Has hearing aids but still impaired hearing	Cannot hear
**OR Vision**				
Parent report	No visual impairment	Visual impairment:<br>No impairment when wearing glasses	Visual impairment:<br>Serious visual impairment that remains despite glasses<br>OR<br>Admitted to centre for visual impairments	Visual impairment:<br>Bilateral blindness

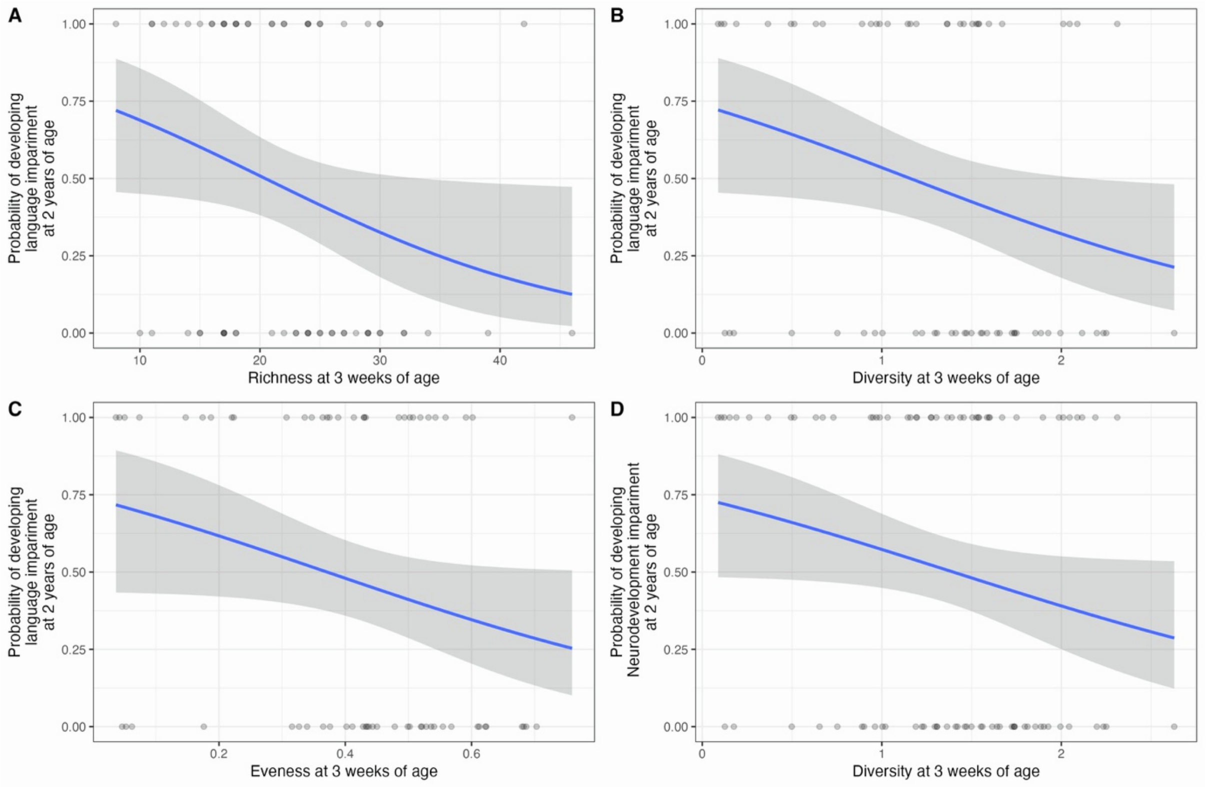

Figure S1. Logistic regression modeling the probability of developing language impairment at 2 years of age as a function of microbial richness at 3 weeks of age (A), microbial diversity (Shannon-index) at 3 weeks of age (B), and microbial evenness at 3 weeks of age (C). And the probability of developing overall neurodevelopment impairment at 2 years of age as a function of microbial diversity at 3 weeks of age (D). Related to Figure 2.

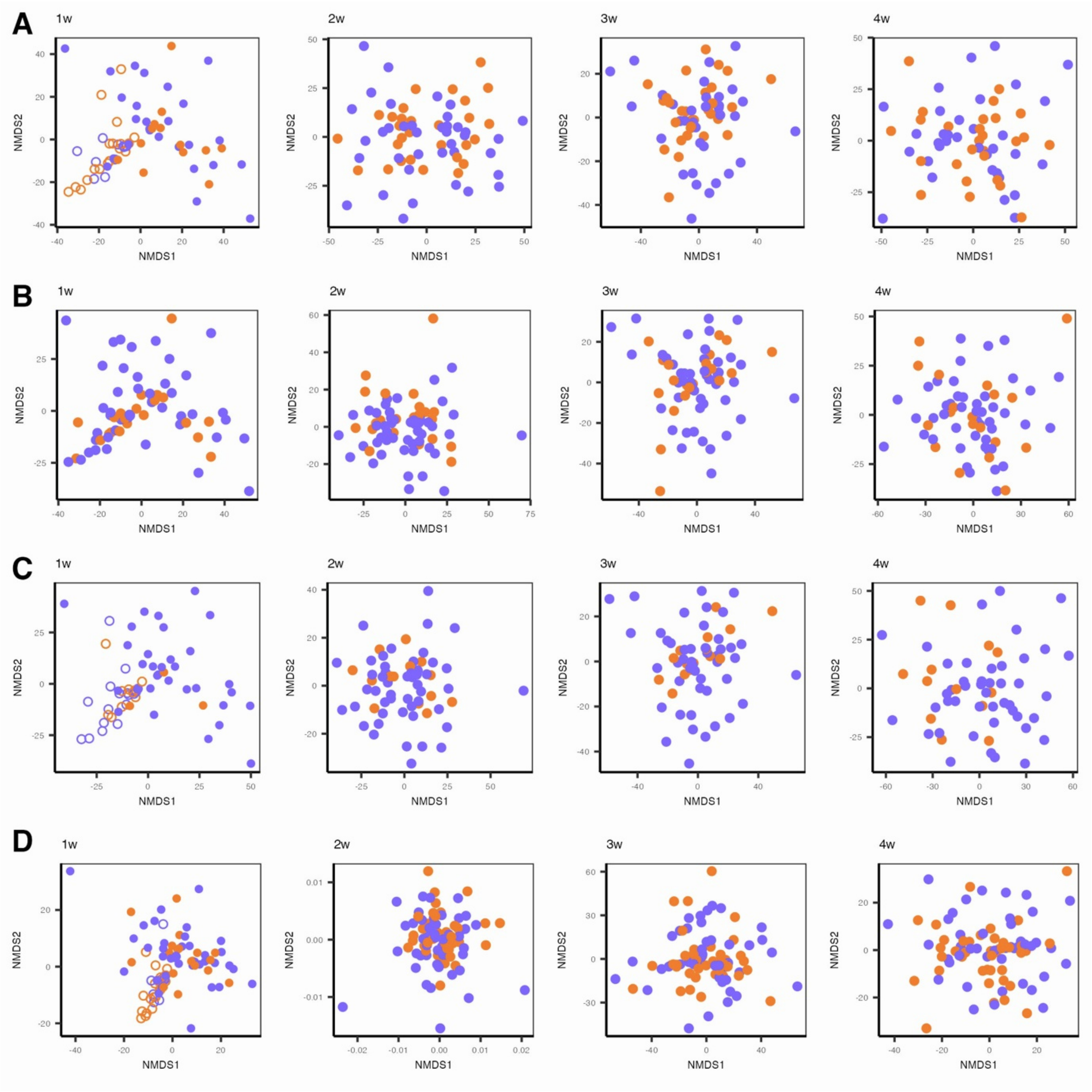

Figure S2. Non-metric multidimensional scaling (NMDS) of the bacterial community composition from 1week (w) to 4 weeks (w) of life across ELBW-EPT infants with normal vs impaired language (A), cognition (B), and motor (C) development, overall neurodevelopment (D) at 2 years of age. Purple: normal development, orange: impaired development. At 1w, the open and filled circles denote inclusion site. Gestational age was a potential confounder for motor and ndi 1w, but the data is not shown. Related to Figure 3.

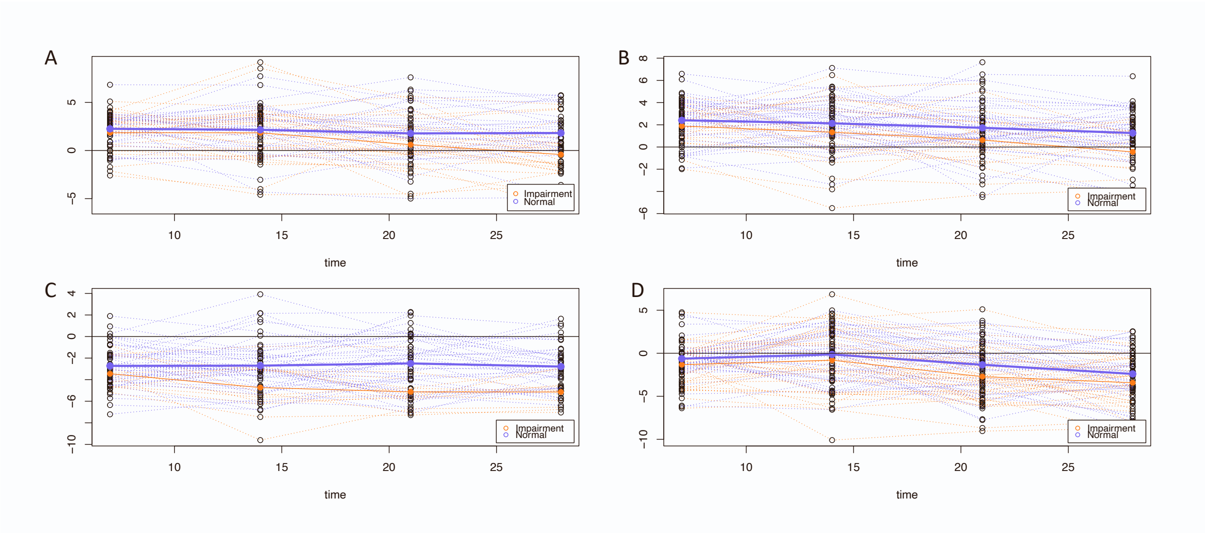

Figure S3. Microbial signature trajectories. Mean relative abundance of the microbial signature discriminative for normal and impaired groups for language development (A), cognition development (B), motor development (C), and overall neurodevelopment impairment (D). Related to Figure 4.
